# Shotgun Proteomic Analysis on the Diapause and Non-Diapause Eggs of Domesticated Silkworm *Bombyx mori*

**DOI:** 10.1371/journal.pone.0060386

**Published:** 2013-04-08

**Authors:** Lanfen Fan, Jianrong Lin, Yangsheng Zhong, Jingyi Liu

**Affiliations:** 1 College of Animal Science, South China Agricultural University, Guangzhou, People's Republic of China; 2 Key Laboratory of Ecology and Environmental Science in Guangdong Higher Education, Guangdong Provincial Key Laboratory for Healthy and Safe Aquaculture, College of Life Science, South China Normal University, Guangzhou, People's Republic of China; Lawrence Berkeley National Laboratory, United States of America

## Abstract

To clarify the molecular mechanisms of silkworm diapause, it is necessary to investigate the molecular basis at protein level. Here, the spectra of peptides digested from silkworm diapause and non-diapause eggs were obtained from liquid chromatography tandem mass spectrometry (LC-MS/MS) and were analyzed by bioinformatics methods. A total of 501 and 562 proteins were identified from the diapause and non-diapause eggs respectively, of which 309 proteins were shared commonly. Among these common-expressed proteins, three main storage proteins (vitellogenin precursor, egg-specific protein and low molecular lipoprotein 30 K precursor), nine heat shock proteins (HSP19.9, 20.1, 20.4, 20.8, 21.4, 23.7, 70, 90-kDa heat shock protein and heat shock cognate protein), 37 metabolic enzymes, 22 ribosomal proteins were identified. There were 192 and 253 unique proteins identified in the diapause and non-diapause eggs respectively, of which 24 and 48 had functional annotations, these unique proteins indicated that the metabolism, translation of the mRNA and synthesis of proteins were potentially more highly represented in the non-dipause eggs than that in the diapause eggs. The relative mRNA levels of four identified proteins in the two kinds of eggs were also compared using quantitative reverse transcription PCR (qRT-PCR) and showed some inconsistencies with protein expression. GO signatures of 486 out of the 502 and 545 out of the 562 proteins identified in the diapause and non-diapause eggs respectively were available. In addition, Kyoto Encyclopedia of Genes and Genomes (KEGG) pathway analysis showed the Metabolism, Translation and Transcription pathway were potentially more active in the non-dipause eggs at this stage.

## Introduction

Diapause is a special physiological state of arrested development by many insects to avoid unfavorable environments such as low temperature, drought or food shortage and to synchronize their life cycles to these changes [1], [2]. Diapause is widespread among insects and can occur at any stage of the life cycle, i.e., adult, pupa, larva or egg, and each species enters diapause at fixed stages. Diapause is endogenously controlled, and this dormancy typically begins well before conditions become too harsh to support normal growth and development.

In the silkworm *Bombyx mori*, the development of diapause-destined embryos is arrested during the G2 cell cycle stage immediately after formation of the cephalic lobe and telson and sequential segmentation of the mesoderm [3]. In *Bombyx mori*, embryonic diapause is determined by a diapause hormone that is secreted by the suboesophageal ganglion (SG) of the mother moth during the pupal period to act on her developing ovaries and is responsible for induction of embryonic diapause of the silkworm, *Bombyx mori* [4], [5]. The termination of diapause requires a low temperature of 5°C for 2–3 months [6], various stimuli can also artificially prevent or terminate diapause, such as HCl, physical stress, corona discharge, or higher oxygen pressure [7]. Once diapause terminates, the embryos are competent to resume development at 25°C and cells enter the M phase; Cell division in the embryos then resumes [3].

However, the molecular mechanisms involved in generating, maintaining, and breaking diapause have not yet been fully elucidated [1]. Currently, only a few studies have elaborated the molecular mechanisms that regulated diapause [8], [9], [10], [11]. Most of these studies have mentioned the molecular regulation of diapause in the larvae, pupae and adults stage. However, the molecular regulation of embryonic diapause is still unclear, perhaps because of the difficulty of extracting the limited amounts of RNA from a developmental stage that has relatively little tissue and large amounts of protein and lipid.

The gene expression profiles linked with diapause have been researched in many insects, such as northern malt fly species [12], [13], flesh fly [14], Chinese oak silkworm [15], Allonemobius socius [16], Drosophila melanogaster [17], Colorado potato beetle [18]. Nevertheless, gene expression profile alone is not sufficient to explain gene functions, and mRNA levels incompletely correlate with the protein levels [19], [20] because of the alternative splicing and dynamics of gene translation. Therefore, with the gradual completion of genome sequencing of insect model organisms, proteomics has become the focal point in entomological research including the diapause mechanism. For a long time, two-dimensional gel electrophoresis (2-DE) combined with mass spectrometry (MS) has been frequently used in insect proteomic research [21], [22]. Another relevant approach in proteomics for large-scale characterization of proteome profiles is shotgun proteomics, which is based on in-gel or gel free digestion of protein mixtures followed by liquid chromatography tandem mass spectrometry (LC-MS/MS) separation, MS detection and database searching, and provides an extremely sensitive and high-throughput approach to determine the proteome components in a complex biological sample. This approach has been widely used in many organisms, such as *Bombyx mori* [23], [24], [25], *Homo sapiens* [26], [27] and *Orientia tsutsugamushi* [28], [29].

Following the rapid development of proteomics and bioinformatics approaches, the credibility of results is greatly promoted. In this study, we utilized the shotgun LC–MS/MS approach combined with bioinformatics analysis to illuminate the differences among protein identification profiles of the diapause and non-diapause eggs of the silkworm and to find valuable clues regarding the molecular regulation of diapause at the early stage of embryogenesis in the silkworm.

## Materials and Methods

### Silkworm Rearing and Sample Collection

Bivoltine silkworm strain 932 was protected at 20°C with half on a short-day photoperiod (8-h-light:16-h-dark for the generation of non-diapause eggs) and half on a long-day photoperiod (16-h-light:8-h-dark for the generation of diapause eggs) separately during the hatching period of silkworm eggs. Larvae were reared on fresh mulberry leaves under an environment of 24 to 25°C and 85% relative humidity. Pupae were kept at 25°C. After female moths emerged, they copulated with males (usually within 5 h) and then laid the eggs at 25°C. Diapause and non-diapause eggs which laid by 30 female moths seperately within 1 h were collected to obtain synchronously developing egg batches. All the diapause and non-diapause eggs were collected 24 h after oviposition and divided into 0.1 g each sample (corresponding to about 200 eggs, three copies). All samples were stored at −80°C until use.

### Sample Preparation and SDS-PAGE Separation

Samples of diapause eggs (D) and non-diapause eggs (ND) were mechanically homogenized on ice for 10 min in 10 µL lyses buffer (comprising 8 M urea, 2 M thiourea, 2% CHAPS, 20 mM Tris–HCl, 30 mM DTT) per 1 mg tissue. The samples were then sonicated in an ice-bath for five circles and each circle contained a 30 s sonication with a 30 s interval. The samples were then centrifuged at 12,000×g at 4°C for 15 min. The supernatants were then collected and were centrifuged again and the resultant supernatants were stored at −20°C for further study. The concentrations of protein samples were determined by the Bradford methods [30].The samples were boiled for 2 min and centrifuged at 12,000×g for 10 min before they were subjected to SDS-PAGE, using a 5% stacking gel and a 12.5% resolving gel. For each sample, a total of 300 µg protein was separated using SDS-PAGE on three lanes with 100 µg each lane. The gels were stained with Coomassie Brilliant Blue (CBB) R250 (Sigma, USA) after electrophoresis.

### In-gel Digestion

**Figure 1:**
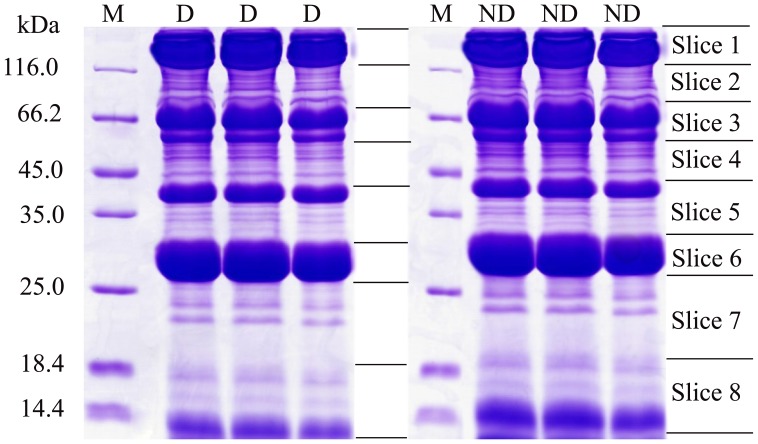
SDS-PAGE patterns of the diapause (D) and non-diapause (ND) eggs protein samples respectively. The samples were separated by 12.5% resolving gel in triplicate. The three batches for both ND and D were pooled into two sets of eight prior to in-gel digest. Then eight slices were combined to four groups for each sample (comprising slices 1 and 2, slices 3 and 4, slices 5 and 6, slices 7 and 8 for both ND and D) prior to the LC separation and MS detection.

Each gel lane was manually cut into 8 bands according to the deepness of Coomassie staining (Fig. 1). The gel bands were sliced into 1×1 mm pieces and subjected to in-gel tryptic digestion which was essentially carried out as described by Shevchenko et al. [31]. Briefly, the gel pieces were rinsed thrice using Milli-Q water (Millipore) and destained twice with 25 mM NH_4_HCO_3_ in 50% acetonitrile (ACN, Amersham) at 37°C until the color depigmented completely. The dried gels were incubated with 50 mM Tris[2-carboxyethyl]phosphine (TCEP, Sigma) in 25 mM NH_4_HCO_3_ at 56°C for 1 h to reductively cleave the disulfide bonds of proteins and then the resulting sulfhydryl functional groups were alkylated by 100 mM iodoacetamide (IAA, Amersham) in 25 mM NH_4_HCO_3_ at room temperature in the dark for 0.5 h. Gel pieces were washed twice with 25 mM ammonium bicarbonate in 50% acetonitrile solution, dehydrated twice with 100% acetonitrile, and dried in a vacuum centrifuge. Subsequently, the proteins were digested with 20 ng/µL modified trypsin (Sigma) for 20 h at 37°C.The resulting peptides were extracted twice from the gel pieces with 5% trifluoroacetic acid (TFA, Fluka, Milwaukee, WI, USA) in 50% ACN solution. The pooled extracts were evaporated in a vacuum centrifuge (Labconco, Kansas, MO), and resuspended with 0.1% methanoic acid (Sigma). After trypsin digested, 8 slices were combined to 4 groups for each sample (slice 1 and 2, slice 3 and 4, slice 5 and 6, slice 7 and 8) prior to the LC separation and MS detection.

### Shotgun LC-MS/MS Analysis

All digested peptide mixtures were separated by reverse phase (RP) HPLC followed by tandem MS analysis. RP-HPLC was performed on a surveyor LC system (Thermo Finnigan, San Jose, CA). Samples were loaded into a trap column (Zorbax 300SB-C18 peptide traps, 300 µm × 65 mm, Agilent Technologies, Wilmington, DE) first at a 3 µL/min flow rate for peptides enrichment and desalting. After flow-splitting down to about 1.5 µL/min, peptides were transferred to the analytical column (RP-C18, 150 µm × 150 mm, Column Technology, Inc., Fremont, CA) for separation with a 195 min linear gradient from 95% buffer A (0.1% methanoic acid in water) to 50% buffer B (84% ACN, 0.1% methanoic acid in water) at a flow rate of 250 nL/min. The analytical column was regenerated for 20 min with buffer A before loading the next sample. A Finnigan LTQ linear ion trap mass spectrometer equipped with a nanospray source was used for the MS/MS [32], [33] experiment in the positive ion mode. The temperature of the ion transfer capillary was set at 170°C. The spray voltage was 3.0 kV and normalized collision energy was set at 35.0% for MS/MS. The MS analysis was performed with one full MS scan (m/z 400–1800 with a resolution R = 60,000 at m/z 400) followed by 10 MS/MS scans on the 10 most intense ions from the MS spectrum with the dynamic exclusion settings: repeat count 2, repeat duration 30 s, exclusion duration 90 s. Data were acquired in data dependent mode using Xcalibur software (version 2.0.7, Thermo Fisher Scientific).

### Database Search

Database search was carried out against the in-house database [34], with the protein sequences downloaded from NCBInr protein database (http://www.ncbi.nlm.nih.gov/) including the domesticated silkworm (*B.mori*,2224 entries), the wild silkworm (*B. mandarina*,13 entries) and the fruit fly (*D. melanogaster*, 27777 entries), along with predicted *B. mori* protein sequence data (14,623 entries) [35]. _The four raw datasets of ND and D samples were searched against the in-house database on a local server using Turbo SEQUEST software (Bioworks version 3.2, Thermo Finnigan). The mass tolerances of precursor ion and fragmentation ion were set to 1.5 Da and 1.0 Da, respectively. The trypsin-cleavage was at both ends of the protein and two missing cleavage sites were allowed. Only b and y fragment ions were taken into account. Static (carbamidomethyl) modification on cysteine, and variable modifications (oxidation) on methionine were set for all searches. The results for each dataset were stored as.out format files. All the.out files were filtered by Buildsummary software. The protein identification criteria that we used were based on Delta CN (≥0.1) and Xcorr (one charge ≥1.9, two charges ≥2.2, three charges ≥3.75) [36].

### Quantitative RT-PCR

Total RNA was extracted from diapause and non-diapause eggs samples using TRIzol reagent (Invitrogen, Carlsbad, CA, USA) according to the manufacturer's instructions and then reverse-transcribed into cDNA with random primer and M-MLV reverse transcriptase (Promega, Madison, WI, USA). The qRT-PCR was performed in 25 µL reactions with 100 ng reverse transcription product, 200 nM each of the forward and reverse primer, and the SYBR® Premix *Ex Taq*™ (Takara, Tokyo, Japan). The cDNA was amplified in a Rotor-Gene 3000 real-time PCR system (Corbett Research, Sydney, Australia) according to the following program: initial denaturation at 95°C for 10 min, and 40 cycles for amplification at 95°C for 30 s, 56°C for 30 s and 72°C for 30 s followed by an additional steps for melting curve with the increase of temperature from 72 to 95°C at 0.5°C/6 s and 30°C for 30 s. The expression levels were calculated using Rotor-Gene software (version 6.0.19) on the basis of the difference of Ct value by normalizing with the reference gene (*BmactinA3*, accession No.X04507). The diapause-unique gene BGIBMGA001595 (Aliphatic nitrilase, *BmnitA*)and the non-diapause-unique genes BGIBMGA002594 (adenylate kinase 2, *BmADK2)*, BGIBMGA011412 (Isocitrate dehydrogenase, *BmIDH*), BGIBMGA002521 (gamma-glutamyltransferase, *BmGGT*) were chosen for the qRT-PCR. The qRT-PCR was performed using the following primers: *BmnitA* forward, TCGGGAAACATCGCAAGAAC and reverse, GCCGTATCTGGTCGCAAATAC, *BmADK2* forward, ATCACGCTCAAACGGTTCCTT and reverse, AGACGTCATCAGCGGCTTTC, *BmIDH* forward, CGTTGCGACCAGACATAAGGA and reverse, TTCACGGATTCGTGTTCCAA, *BmGGT* forward, GACAGCCTCAAACCCAATCAG and reverse, GCGGCCATAAAGCCATCTC.

### Bioinformatics Analysis

Protein sequences were searched against InterPro member databases using the InterProScan software to identify protein signatures [37]. The compiled RAW outputs were subjected to gene ontology (GO) category analysis using the Web Gene Ontology Annotation Plot (WEGO) [38]. The two groups of datasets were simultaneously subjected to online analysis (http://wego.genomics.org.cn/cgi-bin/wego/index.pl) to conveniently compare them in one graph. The P-values were calculated using Pearson Chi-Square test where available. The proteins identified were classified into cellular component, molecular function and biological process. The pathways related to the identified proteins were predicted by searching against KEGG reference pathway database (http://www.genome.jp/kegg/tool/search_pathway.html) with the available protein sequences.

## Results and Discussion

### Proteome Profiles of the D Compared with ND Samples

**Figure 2:**
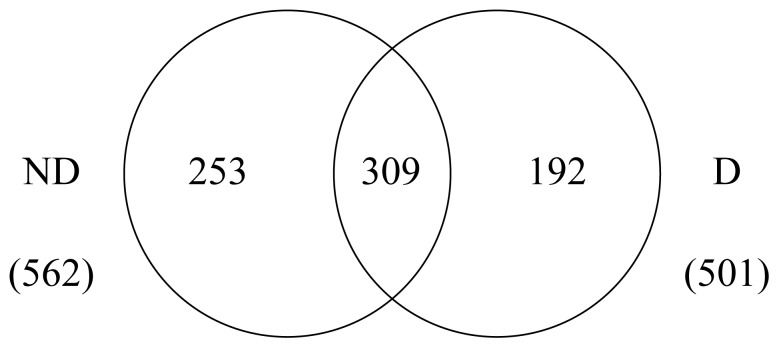
Venn diagram shows the numbers of identified proteins from diapause and non-diapause eggs of the silkworms. Each number with no overlap of circles shows the number of proteins uniquely observed in that sample, while overlapping circle shows the numbers of identified proteins common to two of the analyzes.

The shotgun proteomics strategy, based on proteolytic digestion of complex protein mixtures, peptides LC separation and tandem MS sequencing, has been widely utilized. However, database searching remains the bottleneck for many shotgun proteomics experiments. So in our research, the 4 raw data of each sample sets from LC-MS/MS were subjected to in-house database search using SEQUEST. A total of 501 and 562 proteins were identified from the D and ND respectively (Fig. 2), with 8091 peptides including 1560 unique peptides and 8125 peptides including 1826 unique peptides respectively. The number of common-expressed proteins among the two samples was 309, which was 61.68% and 54.98% of the proteins in the D and ND respectively. Moreover, there were 135 common-expressed proteins with functional annotations (Table S1), the others were predicted proteins from the silkworm genome database. There were 192 and 253 unique proteins identified in the D and ND respectively, of which 24 and 48 had functional annotations (Table 1 and 2).

**Table 1:** Table 1 the unique expressed proteins of D with functional annotation.

Gene ID/GI number	Theor.pI/Mw(kDa)	No. of peptides	No. of unique peptides	Cover percent (%)	Protein description	Unique peptide sequences
BGIBMGA013139 gi|153791691|	7.36/58913.37	1	1	3.28	AMP-activated proteinkinase	K.IADFGLSNM*MMDGEFLR.T
BGIBMGA012151 gi|114053021|	6.62/42674.16	1	1	4.52	ARP1 actin-related protein1-like protein A	R.AQYVLPDGTQLEIGPAR.F
BGIBMGA007039 gi|112983070|	6.06/12248.78	2	1	11.43	BCP inhibitor	K.FADYTPEEQQSR.L
BGIBMGA006850 gi|112983188|	6/55968.01	2	2	2.22	calcineurin A	K.NAEHFSHNSVR.G
gi|160358387|	8.82/59918.81	1	1	2.13	cytochrome P450 CYP6AE9	R.EVMEDYVLLDK.I
gi|160358391|	8.76/59701.51	1	1	2.52	cytochrome P450 CYP6AE7	K.TMGYLFFESTTRR.G
BGIBMGA010671 gi|114053117|	5.38/25082.82	2	2	11.47	eukaryotic translation initiation factor 3 subunit k	K.NNLANLLGGIDDVTLK.H R.ISGFHDSIR.K
BGIBMGA000619 gi|169234934|	4.77/23880.86	2	2	11.57	FK506-binding protein	K.LTIPASLGYGER.G K.LVEEIFQHEDKDK.N
BGIBMGA004040 gi|112983048|	8.51/14345.72	2	1	8.87	hypothetical protein LOC692620	K.LPDLWEELAIK.E
BGIBMGA005301 gi|148298789|	4.25/28434.35	2	2	13.97	hypothetical protein LOC778506	K.IDDSVKPTEVAAATEEK.K K.KDDIAPEDSDIAKPETVPEVK.T
gi|114051870|	6.76/28608.9	1	1	8.33	lutathione transferase o1	-.MTYFHSVNAGVIPPPALTDK.L
BGIBMGA014404 gi|112983100|	4.96/45937.83	1	1	2.38	masquerade-like serine proteinase homolog	R.AGEWDTQNTK.E
BGIBMGA00145 gi|112982906|	6.53/44709.45	1	1	3.71	MAP kinse-ERK kinase	R.IPESILGTITSAVLK.G
BGIBMGA00052 gi|112983290|	5.8/87764.03	1	1	1.68	neutral endopeptidase 24.11	R.VIDLDQASLGLSR.E
gi|182509212|	8.8/51474.1	1	1	2.24	olfactory receptor-like receptor	R.LLLNLLLANR.R
BGIBMGA008164 gi|112983896|	6.33/50013.91	2	2	6.42	putative paralytic peptide-binding protein	K.VGDQQLFLIENR.E R.IPVVSVDDSDTSSYIR.D
BGIBMGA004657 gi|114052160|	6.44/27143	1	1	4.47	proteasome subunit alpha type 6-A	R.GTDAAVVAAQR.K
BGIBMGA000867 gi|112982669|	10.46/28069.61	1	1	4.21	ribosomal protein S2	K.TYAYLTPDLWR.D
BGIBMGA010178 gi|112982956|	11.37/35506.38	1	1	3.59	splicing factor arginine/serine-rich 6	R.VYVGGLPFGVR.E
gi|169234928|	5.06/369030.66	1	1	0.40	titin1	K.PEEANTLRALVDK.I
BGIBMGA000624 gi|148298772|	5.24/380464.73	1	1	0.36	titin2	K.PEGPVIM*REISR.E
BGIBMGA011615 gi|112983266|	8.9/176184.13	2	1	1.03	topoisomerase II	K.FKM*EKLEDDIVLLMSR.R
BGIBMGA001584 gi|160333857|	4.81/29389.15	1	1	5.16	tropomyosin isoform 3	K.LSEASQAADESER.I
gi|112984130|	6.96/33312.27	2	1	4.93	yellow6	R.QNLIPYNFIDDVIR.T

**Table 2:** Table 2 the unique expressed proteins of ND with functional annotation.

Gene ID/GI number	Theor.pI/Mw(kDa)	No. of peptides	No. of unique peptides	Cover percent (%)	Protein description	Unique peptide sequences
gi|114051770|	5.99/43014.71	4	3	11.17	26S proteasome non-ATPase regulatory subunit 13	K.FAVIDVSDFLTK.K R.ALAGAGAVLTAAR.V R.GDNLIQLYNNFLTTFESK.I
BGIBMGA001490 gi|112983564|	4.75/44677.53	3	3	8.21	45 kDa immunophilin FKBP45	K.ALSGGVQIEDLK.L K.TGDSIAFLTNGK.C K.VVMVYYEGR.L
BGIBMGA012298gi|114053253|	7.12/52872.82	2	1	3.31	6-phosphogluconate dehydrogenase	K.NPQLTNLLLDPFFSER.I
BGIBMGA008749 gi|114052224|	8.37/19958.11	1	1	6.32	adenylate cyclase	R.FYADSAATLVR.Y
BGIBMGA011922 gi|114052488|	6.47/40033.17	1	1	3.99	alcohol dehydrogenase	R.IIGVDINPDKFEVAK.K
BGIBMGA007656 gi|148298845|	11.42/20174.53	1	1	7.95	arginine/serine-rich splicing factor 7	R.NPPGFAFVEFEDPR.D
BGIBMGA008670 gi|114052262|	8.31/27604.56	3	2	13.58	ATP synthase	K.KENVLLQLEAAYR.E K.LAAWLDKEVEATENEWNEGR.N
BGIBMGA000622 gi|168823429|	5.93/544514.58	1	1	0.23	Bm kettin	K.TDIIDESVTIK.P
BGIBMGA011218 gi|114052306|	4.77/84822.51	4	3	5.95	Carboxylesterase	K.TETPEQAPSVK.E K.VLETSNNLFGLVIEK.E R.TANIPILVGTTSLEYACER.K
BGIBMGA006916 gi|134254470|	8.54/61666	1	1	5.36	cytochrome P450 18a1	K.LPPGPWGPPVVGYLPFLGVRHKTFLQLAR.N
BGIBMGA003391 gi|114052917|	9.94/12748.96	1	1	8.40	DnaJ domain-containing protein	K.FDSETLANSK.Y
BGIBMGA014398 gi|114052402|	5.49/49150.93	1	1	2.06	eukaryotic translation termination factor 1	K.SQEGSQFVR.G
BGIBMGA007889 gi|114051800|	5.71/36908.18	2	1	8.51	eukaryotic translation initiation factor 3 subunit 2 beta	K.IHSVKEHTHQITDM*QLSRDGTMFVTASK.D
BGIBMGA002526 gi|112983824|	5.34/74917.21	2	1	1.54	ecdysteroid-inducible angiotensin-converting enzyme-related gene product	K.NSIIEKPTDR.E
BGIBMGA008780 gi|112982960|	6.75/26033.48	3	2	6.11	ferritin	K.ALASLYLK.R R.IFFIHR.E
BGIBMGA011438 gi|112983348|	8.72/22373.68	1	1	6.03	glutathione peroxidase	R.HGPNTDPLDLVK.S
BGIBMGA005781 gi|169646838|	5.15/25392.59	1	1	8.07	heat shock protein 25.4	K.IQNLPWDVNSEGSWVYEK.D
BGIBMGA008736 gi|112982982|	5.12/44820.95	3	3	7.80	hemolin	K.EAPAEVLFR.E K.SDFGVASTR.A R.TLATQGEDVTIPCK.A
BGIBMGA004112 gi|114051598|	5.34/69314.08	1	1	2.81	leukotriene A4 hydrolase	R.YLEDLIGGPEVFDDFLR.S
gi|114052571|	6.43/23644.23	2	1	11.82	lysophospholipase	R.HTASLIFLHGLGDTGHGWASTIAGIR.G
BGIBMGA006419 gi|153792270|	6.12/67258.39	3	2	5.39	malate dehydrogenase	K.YCTFNDDIQGTAAVAVAGLLASLR.L R.FAQDHAPVR.T
BGIBMGA004838 gi|114052779|	10.08/48824.97	1	1	3.29	mitochondrial ribosomal protein S5	K.LWKSVTSVSNAGAK.K
BGIBMGA002937 gi|182509194|	6.51/124059.24	1	1	1.82	nitric-oxide synthase like protein	K.QFVSCTVKANKDLGDASAER.S
gi|112984438|	5.55/203642.64	7	6	4.16	ovarian serine protease	K.GPYEQIYK.V K.ISLLHLK.S K.KPLFAVSNTEGFLHR.N K.LTIEQIEQEANHICHYLGFSSAR.T K.VYVEGNYR.C R.TLYTDFDETPLFLR.E
BGIBMGA008602 gi|112984330|	6.26/262354.41	1	1	0.64	p260	R.QPYCGLYGLVKKLSK.Q
BGIBMGA001292 gi|114052687|	5.44/26024.4	1	1	5.75	progesterone receptor membrane component 2	-.MDSPPEVENVQAK.H
BGIBMGA003028 gi|114052034|	8.29/27871.9	1	1	5.62	proteasome beta-subunit	K.LTVEDPVTLEYITR.Y
BGIBMGA013898 gi|114050993|	4.98/26874.64	2	1	5.76	proteasome zeta subunit	K.AIGSGSEGAQQSLK.E
BGIBMGA005994 gi|114052713|	5.92/37494.59	1	1	3.94	proteasome 26S non-ATPase subunit 7	R.DIKDTTVGSLSQR.I
BGIBMGA007169 gi|112983398|	6.33/21828.97	1	1	5.73	ras oncoprotein	K.LVVVGAGGVGK.S
BGIBMGA001800 gi|112982800|	11.66/48110.93	1	1	2.57	ribosomal protein L4	K.IPELPLVVADK.V
BGIBMGA002811 gi|160333861|	9.98/25492.78	1	1	13.24	ribosomal protein L10	R.ATVDDFPLCVHLVSDEYEQLSSEALEAGR.I
BGIBMGA010867 gi|112984078|	10.27/29623.84	6	4	11.41	ribosomal protein S4	K.LGGVYAPR.P K.LRECLPLVIFLR.N K.YALTGNEVLK.I R.ECLPLVIFLR.N
gi|112982661|	10.97/29058.07	1	1	6.72	ribosomal protein S6	R.KGAQEIPGLTDGNVPRR.L
gi|114052054|	5.9/19293.48	1	1	7.69	rotamase Pin1	R.TKEEALDILQEYR.R
BGIBMGA004340 gi|114052803|	8.6/11739.45	1	1	15.32	salivary cysteine-rich peptide	K.SCSEPAAYGGGTGSSSK.H
BGIBMGA007079 gi|114052783|	7.56/51220.85	1	1	4.09	serine hydroxymethyltransferase	K.LLNSNLWEADPELFDIIVK.E
BGIBMGA010212 gi|114051043|	5.28/51672.02	1	1	3.06	serine protease inhibitor serpin	R.EVRIKFSTIIDSLK.K
BGIBMGA006043 gi|114052122|	7.74/33484.08	1	1	4.12	stathmin	R.LTLEQQTAEVYK.A
BGIBMGA011499 gi|112984508|	9.03/50600.85	2	1	2.78	translation initiation factor 2 gamma subunit	R.TTQSNLHQQDLSK.L
BGIBMGA010283 gi|148298831|	7/88104.06	1	1	1.02	tuftelin interacting protein 11	K.M*FPEDILK.E
BGIBMGA005794 gi|151301088|	7.17/22241.48	1	1	5.10	ubiquinone biosynthesis protein COQ7-like protein	R.TLM*QDPNVDK.E
gi|114052396|	6.83/20898.87	3	2	10.38	ubiquitin-conjugating enzyme E2M	K.DLNELNLPK.T K.EAADELQSNR.R
BGIBMGA005628 gi|114050831|	7.52/52953.59	1	1	4.86	uridine 5'-monophosphate synthase	K.KDDTCLIIEDVITSGSSILETVK.D
BGIBMGA013190 gi|114053067|	5.95/20271.21	2	1	5.59	vacuolar protein sorting 29	K.TLASDVHVVR.G
BGIBMGA013964 gi|114050729|	8.46/44007.51	1	1	4.15	vacuolar ATPase subunit C	R.YGLPVNFQAVVM*VPAR.K
BGIBMGA010247 gi|114052088|	8.98/26119.27	1	1	5.75	vacuolar ATP synthase subunit E	R.LELIAQQLLPEIR.N
BGIBMGA004036 gi|112983588|	5.83/65983.72	1	1	1.83	Vlg protein	K.VAVAYGGTAVR.H

### Theoretical Two-dimensional Distribution of the Identified Proteins

**Figure 3:**
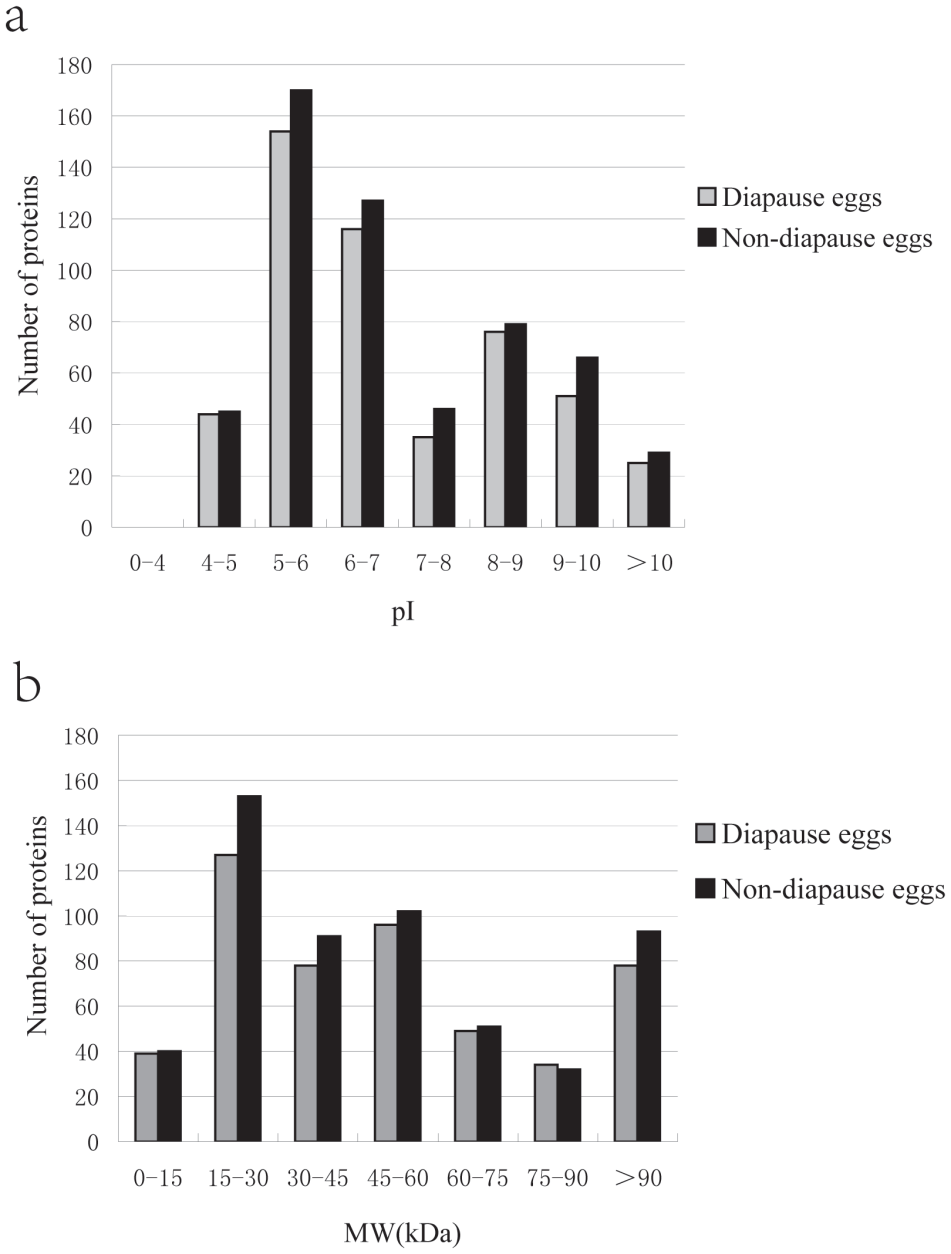
Theoretical pI and Mw distribution of the identified proteins. (a) Distribution of pI. (b) Distribution of Mw. The theoretical isoelectric point (pI) and molecular weight (Mw) of the proteins were calculated using the Compute pI/Mw tool (http://cn.expasy.org/tools/pi_tool.html) according to the predicted amino acid sequences.

The theoretical isoelectric point (pI) and molecular weight (Mw) of the identified proteins were calculated using the Compute pI/Mw tool (http://cn.expasy.org/tools/pi_tool.html) according to the predicted amino acid sequences. It showed that 87.30% of the total proteins were distributed in the range of pI 4–7 and 8–10 (Fig. 3a). About 60.87% of the proteins were distributing in the range of 15–60 kDa (Fig. 3b). In the two kinds of eggs, less than 7.62% of proteins showed pI 7–8. Furthermore, 25 and 29 proteins with higher pI (more than 10), usually difficult to be separated by 2-DE, were also identified from D and ND respectively. It also revealed that the protein distributions were nearly identical in the D and ND samples.

### Profiling of Common-expressed Proteins in the D and ND Samples

On the list of common-expressed proteins of D and ND with annotations, many functional proteins were detected (Table S1). Among these common-expressed proteins we identified, vitellogenin precursor (gi:112983746), egg-specific protein (gi:187281695) and low molecular lipoprotein 30 K precursor (gi:156119320, gi:162461355 and gi:112984502) which comprise the three main storage proteins in the silkworm eggs [39], [40]. The relative concentration of a protein identified by MS in a biological sample is directly related to the number of identified peptides, neglecting possible effects such as the enzymatic digestion constraint, the detection mass range of the mass spectrometer and differential post-translational modifications. Therefore the number of identified unique peptides assembled into a protein may reflect the protein’s relative abundance [25], [41]. So according to the amount of identified unique peptides, vitellogenin precursor, egg-specific protein, low molecular lipoprotein 30 K precursor, and some predicted proteins such as BGIBMGA013342-PA, BGIBMGA004585-PA and BGIBMGA004399-PA are highly represented in the samples. In these predicted proteins, the BGIBMGA013342-PA and BGIBMGA004585-PA were involved in lipid transport (GO: 0006869) with the molecular Function of lipid transporter activity (GO: 0005319), while the BGIBMGA004399-PA was identified to be a low molecular weight lipoprotein which located at the extracellular region (GO: 0005576).These highly-represented proteins were important at the early stage of the embryogenesis [40].

Moreover, many heat shock proteins were identified, such as heat shock protein HSP19.9, HSP20.1, HSP20.4, HSP20.8, HSP23.7, HSP70, HSP90 and heat shock cognate protein. These proteins, HSP19.9, HSP20.1, HSP20.4, HSP20.8, HSP23.7 belong to a family of small heat shock protein (sHSP) which mainly function as molecular chaperones to protect proteins from being denatured during extreme conditions, especially under high temperature stress [42], [43], [44]. The sHSP family is functionally more diverse than other HSPs [29]. Moreover sHSPs can also develop a protection function under other stress conditions, such as cold, drought, oxidation, hypertonic stress, UV, and heavy metals [45], [46], and even under high population densities of organisms [47].The HSPs including sHSPs also play an important role in normal development [44]. However, insect orthologous sHSPs may not be associated with response to environmental stresses and may be involved in basic metabolic processes. Moreover, silkworm sHSPs may play an important role in the development of the germocyte and have functions in immune defense mechanisms [42]. HSP70, heat shock cognate protein,HSP90 and sHSPs are associated with diapause in a number of species [48], [49], [50].But in this study, some HSPs were highly expressed both in diapause and non-diapause eggs, including HSP90, HSP20.8, HSP20.4 and HSP70. A possible explanation is that these HSPs may play an important function in the initial embryos development regardless of diapause or non-diapause.

Furthermore, among the common-expressed proteins with functional annotations, 37 metabolic enzymes were identified (11.97% of the common-expressed proteins with functional annotations). The metabolic enzymes include ADP/ATP translocase, fibroinase, glucose-6-phosphate isomerase, glutamate dehydrogenase, isocitrate dehydrogenase, salivary secreted ribonuclease, transketolase etc. The identification of these essential metabolic enzymes indicated that during embryogenesis stage metabolism was active in the eggs. Many ribosomal proteins (L7, L7A, L9, L10A, L11, L13, L13A, L15, L17, L18, L19, L23A, L38, S3, S5, S7, S8, S9, S15A, S27, S28, and S30) were found in D and ND samples. This abundance of ribosomal proteins on the first day after oviposition might be closely related to protein synthesis during embryogenesis.

In addition, 62 proteins that may also play important roles in the egg development were identified commonly, such as 14-3-3 epsilon protein, antennal binding protein, calreticulin, cellular retinoic acid binding protein, chemosensory protein 11, exuperantia, perilipin, profiling, transferring and etc. 14-3-3 epsilon protein (Bm14-3-3ε) is a member of the 14-3-3 protein family. The 14-3-3 proteins along with partner proteins(for example CDK11, PFTK1, GSK3beta, Chk1) are involved in the regulation of several crucial cellular processes including metabolism, signal transduction, cell development, differentiation, apoptosis, transcription, stress responses and malignant transformation [51], [52], [53], [54], [55], [56]. In this study, we identified Bm14-3-3ε in the D and ND samples. This result indicated that Bm14-3-3ε may play a role in regulating embryonic development of silkworm. Another important protein was translationally controlled tumor protein (TCTP), a highly conserved protein upregulated in various tumours. Hsu et al [57] reported that reducing Drosophila TCTP (dTCTP) levels will reduces its cell size, cell number and organ size. Further more, calreticulin located in the endoplasmic reticulum, has been implicated in many diverse functions, including: regulation of intracellular Ca^2+^ homeostasis, chaperone activity, steroid-mediated gene regulation, and cell adhesion [58]. The highest level of mRNA expression of calreticulin was exhibited in the fat body of *Bombyx mori* [59]. Our result showed that calreticulin was identified during the initial embryogenesis both in D and ND samples, which means that calreticulin is essential during embryonic development.

### Profiling of D-Unique Proteins

The characteristic proteins with functional annotations unique to the D samples are shown in Table 1. Among the D-unique proteins, BCP inhibitor (BCP1, *Bombyx* cysteine proteinase inhibitor) is inhibitory towards the processing of the enzymatically inactive proform of BCP (pro-BCP) to the activated mature BCP but has no effects on trypsin and pepsin, and is highly expressed in the metamorphosis stage of *B.mori* [60]. In our study, BCPI which was exclusively identified in the D samples may be involved in regulating the development of diapause-destined embryos to arrest during the G2 cell cycle stage.

Calcineurin A and FK506-binding protein were other proteins which were uniquely identified in the D sample. Calcineurin A with four EF-hand type calcium-binding structures is localized in the cytoplasm of the pheromone-producing cells and participates in the intracellular signal transduction of PBAN (Pheromone biosynthesis activating neuropeptide) in *B. mori* [61]. The FK506-binding protein (FKBP) belongs to a ubiquitous family of proteins which participates in a variety of pathways, including protein folding, down-regulation of T-cell activation and inhibition of cell-cycle progression [62]. Both Calcineurin A and FK506-binding protein identified in the diapause eggs are related with the Ca^2+^ release, suggesting that in the diapause eggs the regulation of the Ca^2+^ might be more active than in the non-diapause eggs. Other proteins identified in the diapause eggs were cytochrome P450 CYP6AE9, cytochrome P450 CYP6AE7, titin1, titin2, topoisomerase II etc (Table 1).

In the diapause eggs, the cephalic lobe and telson and sequential segmentation of the mesoderm are formed before the embryos enters into diapause [3]. In another words, the development of the diapause eggs are on one hand moving toward embryonic development, while on the other hand preparing to enter diapause after oviposition. Thus, these D-unique proteins may be adapted to this physiological need.

### Profiling of ND-Unique Proteins

Compared with the unique proteins of the D samples, the characteristic proteins unique to the ND samples are shown in Table 2. There were more metabolic enzymes identified in the ND samples than in the D samples, including 6-phosphogluconate dehydrogenase, adenylate cyclase, alcohol dehydrogenase, ATP synthase(subunit B), carboxylesterase, glutathione peroxidase, malate dehydrogenase, ovarian serine protease, serine hydroxylmethyltransferase etc (Table 2). In the non-diapause eggs, the development of the embryos was consistent and without interruption, so the metabolism of the non-diapause eggs was potentially more active than the diapause eggs.

In addition, we also identified eukaryotic translation initiation factor 3 subunit 2 beta, translation initiation factor 2 gamma subunit, eukaryotic translation termination factor 1 and more ribosomal proteins (L4, L10, S4, S6) in the non-diapause eggs. These components are important in regulating translation and protein synthesis, indicating that translation of the mRNA and synthesis of proteins was potentially more active in the non-dipause eggs than that in the diapause eggs. Proteasome beta-subunit, proteasome zeta subunit and proteasome 26S non-ATPase subunit 7 were also identified only in the non-dipause eggs, which play important roles in degrading cytosolic and nuclear proteins previously labeled with ubiquitin molecules [63]. Other proteins identified in the non-diapause eggs were ecdysteroid-inducible angiotensin-converting enzyme-related gene product whose transcription was directly induced by 20-hydroxyecdysone (20E), ubiquitin-conjugating enzyme E2M, stathmin and a ras oncoprotein, all of which are associated with embryonic development [64].

### Quantitative RT-PCR Analysis

**Figure 4:**
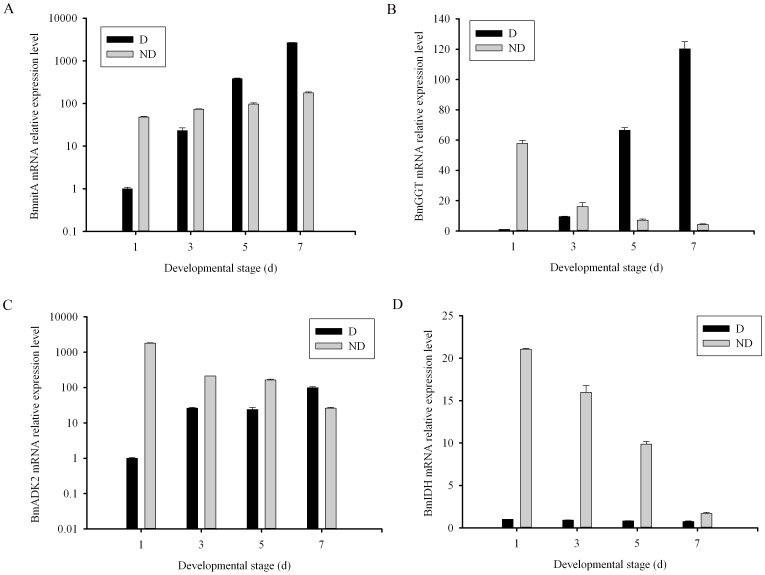
Gene expression level differences of the identified proteins in D and ND eggs of 1, 3, 5, 7 days after oviposition. The mRNA levels of the target genes were normalized by subtracting Ct value of the reference gene, *BmactinA3* (No.X04507). D means diapause and N means non-diapause.The (2^-ΔΔCT^ values represents relative gene expression level. Values are means ± SD (standard deviations).

The dipause-unique gene *BmnitA* and the non-diapause-unique genes *BmADK2*, *BmIDH* and *BmGGT* were selected to detect their relative mRNA levels by qRT-PCR analysis. Relative gene expression levels were showed in Fig. 4. In the diapause eggs, *BmnitA*, *BmADK2* and *BmGGT* mRNA relative expression levels were significantly increased compared with those in non-diapause eggs according to the development stage, while *BmIDH* mRNA relative expression levels were remained constant compared with those in non-diapause eggs according to the development stage, which indicated that *BmIDH* is a possible non-diapause-unique gene. Interestingly at the initial embryogenesis, the mRNA relative expressions of these four genes were all significantly higher in the non-diapause eggs than in the diapause eggs. In the non-diapause eggs, *BmIDH* mRNA relative expression was significantly higher more than 21-fold than that in diapause eggs, *BmnitA* mRNA relative expression was significantly higher more than 21-fold than that in diapause eggs, *BmADK2* mRNA relative expression was significantly higher more than 1795-fold than that in diapause eggs, *BmGGT* mRNA relative expression was significantly higher more than 57-fold than that in diapause eggs. However, the mRNA levels were not always consistent with their protein expressions. In the mRNA level, the expression of these four genes can be detected whether in diapause or non-dipause eggs.

### Gene Ontology Analysis of the Functional Categories

**Figure 5:**
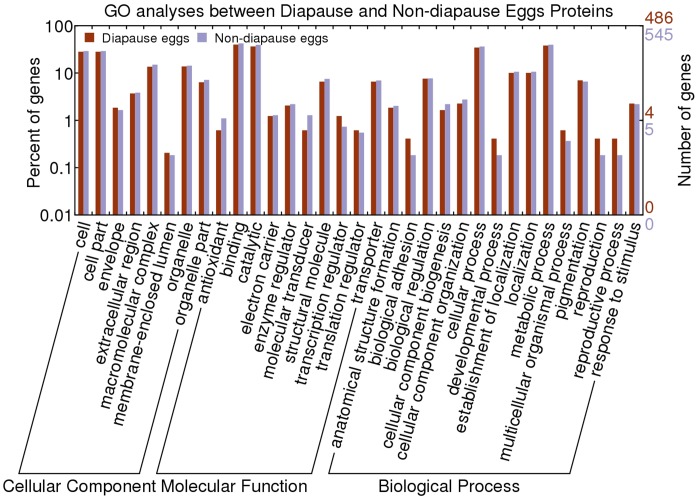
Gene ontology categories for the identified proteins of D and ND. The identified proteins were classified into cellular component, molecular function and biological process by WEGO according to the GO terms. The number of genes is the number of times the GO term is used to annotate genes in the cluster. The left-hand shows its proportion in total genes of related genes with GO terms.

Gene ontology (GO) is now widely used to describe the function of genes and gene products in a standardized format (http://www.geneontology.org) [65]. To understand the functions of the proteins we identified, the protein sequences were queried against the InterPro databases and the resultant proteins were functionally categorized based on universal GO annotation terms using the online GO tool WEGO [38], [66]. GO signatures of 486 out of the 501 and 545 out of the 562 proteins identified in the diapause and non-diapause eggs respectively were available. They were classified into Cellular Component, Molecular Function and Biological Process according to the GO hierarchy using WEGO (Fig. 5).

In the Cellular Component category, proteins mapping to cell, cell part, macromolecular complex and organelle related GO terms were the most abundant, mapping to membrane-enclosed lumen were the fewest. In the subcategory of cell part, 114 and 131 proteins of D and ND respectively were ascribed to intracellular. In the subcategory of organelle, 30 and 35 proteins were separately assigned to membrane-bounded organelle, and 39 and 48 proteins were separately assigned to non-membrane-bounded organelle.

According to the Molecular Function category, most proteins were addressed to binding (194/231 in the D and ND samples respectively) and catalytic activity (177/215 in the D and ND samples respectively), especially the nucleotide, nucleoside, chromatin, ion and nucleic acid binding and hydrolase, oxidoreductase, transferase activities. The groups with much fewer terms (only one protein annotated) include small protein activating enzyme activity, chromatin binding, odorant binding, metal cluster binding, enzyme activator activity, phosphatase regulator activity, transcription repressor activity, transcription initiation factor activity proteins. Moreover, one protein with deaminase activity, two proteins with cyclase activity and one protein with thioredoxin-disulfide reductase activity were annotated functional proteins only in the non-dipause eggs.

Considering the Biological Process category, most of proteins were involved in the metabolic and cellular process. Much fewer proteins were involved in the reproduction, reproductive, biological adhesion and developmental process. In the metabolic process subcategories, most proteins were related to primary metabolic, cellular metabolic and macromolecule metabolic process. In the cellular process subcategories, most proteins were associated with the cellular metabolic process followed by the cell communication process. Besides, only one protein was functionally annotated to each of secondary metabolic process, cellular component disassembly, translational initiation, membrane docking and secretion by cell in the diapause eggs.

GO analysis on the identified proteins presented an overall view on the functional categories of the diapause and non-diapause egg proteomes. In addition, proteins with GO annotation in the diapause eggs were not significantly different (p<0.05) to those in the non-diapause eggs, indicating that the expressed proteins during the early stages of embryonic development have many functional similarities between diapause and non-diapause eggs.

### KEGG Pathway Analysis

KEGG is a large resource contains information for various model organisms about Molecular Interactions, Reaction Networks, Cellular Processes and Human Diseases [67]. In the present study, a total of 689 of 754 proteins were subjected to query against the KEGG reference pathway database and 192 non-redundant pathways were indicated (Table S2) that most of them were related to Metabolism and Organism Systems. Other pathways such as the Genetic Information Processing, Environmental Information Processing, Cellular Processes and Human Diseases were also detected. 42 and 29 KEGG pathways were involve only in the diapause eggs unique proteins and non-diapause eggs unique proteins, respectively (Table 3, 4).

**Table 3:** Table 3 KEGG pathways of diapause unique proteins.

Annotation	Total Genes
**Metabolism**	
Lipid Metabolism; Steroid hormone biosynthesis	3
Lipid Metabolism; Linoleic acid metabolism	3
Xenobiotics Biodegradation and Metabolism; Caprolactam degradation	20
Lipid Metabolism; Biosynthesis of unsaturated fatty acids	14
**Environmental Information Processing**	
Signal Transduction; ErbB signaling pathway	20
Signal Transduction; Phosphatidylinositol signaling system	20
Signal Transduction; mTOR signaling pathway	20
Signal Transduction; VEGF signaling pathway	40
**Cellular Processes**	
Cell Growth and Death; Cell cycle - Caulobacter	20
Transport and Catabolism; Regulation of autophagy	20
Cell Communication; Focal adhesion	21
Cell Motility; Regulation of actin cytoskeleton	21
**Genetic Information Processing**	
Folding, Sorting and Degradation; Sulfur relay system	20
**Organismal Systems**	
Development; Dorso-ventral axis formation	20
Development; Axon guidance	21
Development; Osteoclast differentiation	40
Immune System; Complement and coagulation cascades	9
Immune System; Toll-like receptor signaling pathway	20
Environmental Adaptation; Plant-pathogen interaction	20
Immune System; Natural killer cell mediated cytotoxicity	40
Immune System; T cell receptor signaling pathway	40
Immune System; B cell receptor signaling pathway	40
Immune System; Fc epsilon RI signaling pathway	20
Immune System; Leukocyte transendothelial migration	1
Endocrine System; Adipocytokine signaling pathway	39
**Human Diseases**	
Pathogenic Escherichia coli infection	1
Infectious Diseases; Shigellosis	1
Infectious Diseases; Hepatitis C	19
Infectious Diseases; Measles	19
Cancers; Pathways in cancer	20
Cancers; Colorectal cancer	20
Cancers; Renal cell carcinoma	20
Cancers; Pancreatic cancer	20
Cancers; Endometrial cancer	20
Cancers; Glioma	20
Cancers; Prostate cancer	20
Cancers; Thyroid cancer	20
Cancers; Melanoma	20
Cancers; Bladder cancer	20
Cancers; Chronic myeloid leukemia	20
Cancers; Acute myeloid leukemia	20
Cancers; Non-small cell lung cancer	20

**Table 4:** KEGG pathways of non-diapause unique proteins.

Annotation	Total Genes
**Metabolism**	
Lipid Metabolism; Fatty acid biosynthesis	18
Metabolism of Cofactors and Vitamins; Ubiquinone and other terpenoid-quinone biosynthesis	19
Amino Acid Metabolism; Phenylalanine metabolism	6
Metabolism of Other Amino Acids; Selenocompound metabolism	40
Metabolism of Other Amino Acids; Cyanoamino acid metabolism	35
Lipid Metabolism; Arachidonic acid metabolism	51
Carbohydrate Metabolism; Glyoxylate and dicarboxylate metabolism	20
Metabolism of Terpenoids and Polyketides; Terpenoid backbone biosynthesis	20
Biosynthesis of Other Secondary Metabolites; Indole alkaloid biosynthesis	6
Biosynthesis of Other Secondary Metabolites; Isoquinoline alkaloid biosynthesis	6
Biosynthesis of Other Secondary Metabolites; Betalain biosynthesis	6
Metabolism of Terpenoids and Polyketides; Insect hormone biosynthesis	37
**Genetic Information Processing**	
Translation; RNA transport	20
Translation; mRNA surveillance pathway	20
Transcription; Spliceosome	20
**Environmental Information Processing**	
Signaling Molecules and Interaction; Cytokine-cytokine receptor interaction	2
Signal Transduction; Hedgehog signaling pathway	20
**Cellular Processes**	
Cell Growth and Death; Meiosis - yeast	20
Transport and Catabolism; Endocytosis	19
**Organismal Systems**	
Immune System; RIG-I-like receptor signaling pathway	20
Sensory System; Olfactory transduction	20
Sensory System; Taste transduction	20
Sensory System; Phototransduction - fly	20
Excretory System; Aldosterone-regulated sodium reabsorption	19
Excretory System; Endocrine and other factor-regulated calcium reabsorption	58
Digestive System; Carbohydrate digestion and absorption	19
**Human Diseases**	
Infectious Diseases; Bacterial invasion of epithelial cells	19
Infectious Diseases; Toxoplasmosis	1
Infectious Diseases; Amoebiasis	20

The metabolism pathways in non-diapause eggs were more highly represented than which in diapause eggs. The amino acid metabolism, carbohydrate metabolism and biosynthesis of other secondary metabolites was more highly represented in the non-diapause eggs, meanwhile the lipid metabolism pathway such as steroid hormone biosynthesis, linoleic acid metabolism and biosynthesis of unsaturated fatty acids was more highly represented in the diapause eggs. The translation and transcription pathway such as RNA transport, mRNA surveillance pathway and spliceosome were only detected in the non-diapause eggs. The sensory system (olfactory transduction, taste transduction and phototransduction), excretory system and digestive system were also detected in the non-diapause eggs, which indicated that the embryonic development was in progress.

ErbB signaling pathway, phosphatidylinositol signaling system, mTOR signaling pathway and VEGF signaling pathway which belonged to signal transduction pathway were more active in the diapause eggs. ErbB signaling regulates diverse cellular functions, such as proliferation, migration, differentiation and survival/death, and participates in various developmental processes during both invertebrate and vertebrate early embryogenesis [68], [69]. Interestingly, development and immune system pathways were also more represented in the diapause eggs, maybe the trigger of immune system pathway was the self-protection of diapause eggs during the long diapause stage.

### Conclusions

This study provides a catalogue and an initial analysis of the proteomes of the diapause and non-diapause eggs during embryogenesis of the silkworm by shotgun proteomic analysis. Unique proteins identified in the two kinds of eggs and common proteins shared by each other were identified and analyzed. The relative mRNA levels of four identified proteins in the two kinds of eggs were also compared using qRT-PCR and showed some inconsistencies with protein expression. GO analysis of these proteins also provided us with a global view of their functions. In addition, KEGG pathway analysis showed the Metabolism, Translation and Transcription pathway were potentially more highly represented in the non-dipause eggs at this stage. These results will also help further research on finding the diapause-associated proteins during egg development of silkworm. However, the shotgun proteomic analysis has some shortcomings, for example, it is too complex for protein assembly and it mainly depends on bioinformatic methods. With the development of genomics and bioinformatics, the shotgun LC-MS/MS will be a promising strategy in proteomics research.

## Supporting Information

Table S1 **The common-expressed proteins of D and ND with functional annotation.**
(DOC)

Table S2 **KEGG pathways of all identified proteins.**
(DOC)

## References

[1] Denlinger DL (2002) Regulation of diapause. Annu Rev Entomol 47: 93–122. 11729070 10.1146/annurev.ento.47.091201.145137

[2] Taylor F (1986) Insect life histories: seasonal adaptations of insects. Science 232: 1152. 10.1126/science.232.4754.1152-a 17754501

[3] Nakagaki M, Takei R, Nagashima E, Yaginuma T (1991) Cell cycles in embryos of the silkworm, *Bombyx mori*: G2-arrest at diapause stage Development Genes and Evolution. 200: 223–229. 10.1007/BF00361341 28305970

[4] Yamashita O (1996) Diapause hormone of the silkworm, *Bombyx mori*: Structure, gene expression and function. Journal of Insect Physiology 42: 669–679.

[5] Xu W-H, Sato Y, Ikeda M, Yamashita O (1995) Molecular characterization of the gene encoding the precursor protein of diapause hormone and pheromone biosynthesis activating neuropeptide (DH-PBAN) of the silkworm, *Bombyx mori* and its distribution in some insects. Biochimica et Biophysica Acta (BBA) - Gene Structure and Expression 1261: 83–89. 7893764 10.1016/0167-4781(94)00238-x

[6] Yaginuma T, Kobayashi M, Yamashita O (1990) Distinct effects of different low temperatures on the induction of NAD-sorbitol dehydrogenase activity in diapause eggs of the silkworm, *Bombyx mori* . Journal of Comparative Physiology B: Biochemical, Systemic, and Environmental Physiology 160: 277–285.

[7] Sonobe H, Matsumoto A, Fukuzaki Y, Fujiwara S (1979) Carbohydrate metabolism and restricted oxygen supply in the eggs of the silkworm, *Bombyx mori* . Journal of Insect Physiology 25: 381–388.

[8] Macrae TH (2010) Gene expression, metabolic regulation and stress tolerance during diapause. Cell Mol Life Sci. 10.1007/s00018-010-0311-0 PMC11115916 20213274

[9] Hong SM, Nho SK, Kim NS, Lee JS, Kang SW (2006) Gene expression profiling in the silkworm, *Bombyx mori*, during early embryonic development. Zoolog Sci 23: 517–528. 16849839 10.2108/zsj.23.517

[10] Robich RM, Rinehart JP, Kitchen LJ, Denlinger DL (2007) Diapause-specific gene expression in the northern house mosquito, *Culex pipiens L.*, identified by suppressive subtractive hybridization. J Insect Physiol 53: 235–245. 17098250 10.1016/j.jinsphys.2006.08.008 PMC1894908

[11] Fujiwara Y, Shindome C, Takeda M, Shiomi K (2006) The roles of ERK and P38 MAPK signaling cascades on embryonic diapause initiation and termination of the silkworm, *Bombyx mori* . Insect Biochem Mol Biol 36: 47–53. 16360949 10.1016/j.ibmb.2005.10.005

[12] Kankare M, Salminen T, Laiho A, Vesala L, Hoikkala A (2010) Changes in gene expression linked with adult reproductive diapause in a northern malt fly species: a candidate gene microarray study. BMC Ecol 10: 3. 20122138 10.1186/1472-6785-10-3 PMC2822739

[13] Li A, Rinehart JP, Denlinger DL (2009) Neuropeptide-like precursor 4 is uniquely expressed during pupal diapause in the flesh fly. Peptides 30: 518–521. 19007830 10.1016/j.peptides.2008.10.008

[14] Rinehart JP, Robich RM, Denlinger DL (2010) Isolation of diapause-regulated genes from the flesh fly, *Sarcophaga crassipalpis* by suppressive subtractive hybridization. J Insect Physiol 56: 603–609. 20026067 10.1016/j.jinsphys.2009.12.007

[15] Li YP, Xia RX, Wang H, Li XS, Liu YQ, et al (2009) Construction of a full-length cDNA Library from Chinese oak silkworm pupa and identification of a KK-42-binding protein gene in relation to pupa-diapause termination. Int J Biol Sci 5: 451–457. 19564928 10.7150/ijbs.5.451 PMC2702828

[16] Reynolds JA, Hand SC (2009) Embryonic diapause highlighted by differential expression of mRNAs for ecdysteroidogenesis, transcription and lipid sparing in the cricket Allonemobius socius. Journal of Experimental Biology 212: 2075–2084. 19525434 10.1242/jeb.027367 PMC2702454

[17] Baker DA, Russell S (2009) Gene expression during Drosophila melanogaster egg development before and after reproductive diapause. BMC Genomics 10: 242. 19463195 10.1186/1471-2164-10-242 PMC2700134

[18] Yocum GD, Rinehart JP, Chirumamilla-Chapara A, Larson ML (2009) Characterization of gene expression patterns during the initiation and maintenance phases of diapause in the Colorado potato beetle, *Leptinotarsa decemlineata* . J Insect Physiol 55: 32–39. 18992752 10.1016/j.jinsphys.2008.10.003

[19] Chen G, Gharib TG, Huang CC, Taylor JM, Misek DE, et al (2002) Discordant protein and mRNA expression in lung adenocarcinomas. Mol Cell Proteomics 1: 304–313. 12096112 10.1074/mcp.m200008-mcp200

[20] Gygi SP, Rochon Y, Franza BR, Aebersold R (1999) Correlation between protein and mRNA abundance in yeast. Molecular and Cellular Biology 19: 1720–1730. 10022859 10.1128/mcb.19.3.1720 PMC83965

[21] Li A, Denlinger DL (2009) Pupal cuticle protein is abundant during early adult diapause in the mosquito *Culex pipiens* . J Med Entomol 46: 1382–1386. 19960684 10.1603/033.046.0618

[22] Zhou ZH, Yang HJ, Chen M, Lou CF, Zhang YZ, et al (2008) Comparative proteomic analysis between the domesticated silkworm (*Bombyx mori*) reared on fresh mulberry leaves and on artificial diet. J Proteome Res 7: 5103–5111. 18998723 10.1021/pr800383r

[23] Yang HJ, Zhou ZH, Zhang HR, Chen M, Li JY, et al (2010) Shotgun proteomic analysis of the fat body during metamorphosis of domesticated silkworm (*Bombyx mori*). Amino Acids 38: 1333–1342. 19730979 10.1007/s00726-009-0342-8

[24] Li J, Hosseini Moghaddam SH, Chen X, Chen M, Zhong B (2010) Shotgun strategy-based proteome profiling analysis on the head of silkworm *Bombyx mori*. Amino Acids. 10.1007/s00726-010-0517-3 20198493

[25] Li JY, Moghaddam SHH, Chen JE, Chen M, Zhong BX (2010) Shotgun proteomic analysis on the embryos of silkworm *Bombyx mori* at the end of organogenesis. Insect Biochemistry and Molecular Biology 40: 293–302. 20138214 10.1016/j.ibmb.2010.01.008

[26] Klein LL, Jonscher KR, Heerwagen MJ, Gibbs RS, McManaman JL (2008) Shotgun proteomic analysis of vaginal fluid from women in late pregnancy. Reprod Sci 15: 263–273. 18421021 10.1177/1933719107311189

[27] Lee CL, Hsiao HH, Lin CW, Wu SP, Huang SY, et al (2003) Strategic shotgun proteomics approach for efficient construction of an expression map of targeted protein families in hepatoma cell lines. Proteomics 3: 2472–2486. 14673797 10.1002/pmic.200300586

[28] Ogawa M, Shinkai-Ouchi F, Matsutani M, Uchiyama T, Hagiwara K, et al (2009) Shotgun proteomics of *Orientia tsutsugamushi* . Clinical Microbiology and Infection 15: 239–240. 19438636 10.1111/j.1469-0691.2008.02157.x

[29] Li Z-W, Li X, Yu Q-Y, Xiang Z-H, Kishino H, et al. (2009) The small heat shock protein (sHSP) genes in the silkworm, *Bombyx mori*, and comparative analysis with other insect sHSP genes. Bmc Evolutionary Biology 9. 10.1186/1471-2148-9-215 PMC2745388 19715580

[30] Bradford MM (1976) A rapid and sensitive method for the quantitation of microgram quantities of protein utilizing the principle of protein-dye binding. Anal Biochem 72: 248–254. 942051 10.1016/0003-2697(76)90527-3

[31] Shevchenko A, Tomas H, Havlis J, Olsen JV, Mann M (2006) In-gel digestion for mass spectrometric characterization of proteins and proteomes. Nature Protocols 1: 2856–2860. 17406544 10.1038/nprot.2006.468

[32] Li JY, Chen X, Fan W, Moghaddam SHH, Chen M, et al (2009) Proteomic and Bioinformatic Analysis on Endocrine Organs of Domesticated Silkworm, *Bombyx mori L*. for a Comprehensive Understanding of Their Roles and Relations. Journal of Proteome Research 8: 2620–2632. 19382758 10.1021/pr8006123

[33] Li JY, Chen X, Moghaddam SHH, Chen M, Wei H, et al (2009) Shotgun proteomics approach to characterizing the embryonic proteome of the silkworm, *Bombyx mori*, at labrum appearance stage. Insect Molecular Biology 18: 649–660. 19754742 10.1111/j.1365-2583.2009.00903.x

[34] Li J-y, Moghaddam SHH, Chen J-e, Chen M, Zhong B-x (2010) Shotgun proteomic analysis on the embryos of silkworm *Bombyx mori* at the end of organogenesis. Insect Biochem Mol Biol 40: 293–302. 20138214 10.1016/j.ibmb.2010.01.008

[35] Xia QY, Wang J, Zhou ZY, Li RQ, Fan W, et al (2008) The genome of a lepidopteran model insect, the silkworm *Bombyx mori* . Insect Biochemistry and Molecular Biology 38: 1036–1045. 19121390 10.1016/j.ibmb.2008.11.004

[36] Washburn MP, Wolters D, Yates JR (2001) Large-scale analysis of the yeast proteome by multidimensional protein identification technology. Nature Biotechnology 19: 242–247. 10.1038/85686 11231557

[37] Zdobnov EM, Apweiler R (2001) InterProScan - an integration platform for the signature-recognition methods in InterPro. Bioinformatics 17: 847–848. 11590104 10.1093/bioinformatics/17.9.847

[38] Ye J, Fang L, Zheng HK, Zhang Y, Chen J, et al (2006) WEGO: a web tool for plotting GO annotations. Nucleic Acids Research 34: W293–W297. 16845012 10.1093/nar/gkl031 PMC1538768

[39] Maki N, Yamashita O (1997) Purification and characterization of a protease degrading 30 kDa yolk proteins of the silkworm, *Bombyx mori* . Insect Biochem Mol Biol 27: 721–728. 9443372 10.1016/s0965-1748(97)00050-7

[40] Zhu J, Indrasith LS, Yamashita O (1986) Characterization of vitellin, egg-specific protein and 30 kDa protein from *Bombyx* eggs, and their fates during oogenesis and embryogenesis. Biochimica et Biophysica Acta (BBA) - General Subjects 882: 427–436.

[41] Jin S, Daly DS, Springer DL, Miller JH (2008) The effects of shared peptides on protein quantitation in label-free proteomics by LC/MS/MS. Journal of Proteome Research 7: 164–169. 18001079 10.1021/pr0704175

[42] Li ZW, Li X, Yu QY, Xiang ZH, Kishino H, et al. (2009) The small heat shock protein (sHSP) genes in the silkworm, *Bombyx mori*, and comparative analysis with other insect sHSP genes. Bmc Evolutionary Biology 9: -. 10.1186/1471-2148-9-215 PMC2745388 19715580

[43] Van Montfort R, Slingsby C, Vierling E (2001) Structure and function of the small heat shock protein/alpha-crystallin family of molecular chaperones. Fibrous Proteins: Amyloids, Prions and Beta Proteins 59: 105–156. 10.1016/s0065-3233(01)59004-x 11868270

[44] Sun Y, MacRae TH (2005) Small heat shock proteins: molecular structure and chaperone function. Cell Mol Life Sci 62: 2460–2476. 16143830 10.1007/s00018-005-5190-4 PMC11138385

[45] Waters ER, Aevermann BD, Sanders-Reed Z (2008) Comparative analysis of the small heat shock proteins in three angiosperm genomes identifies new subfamilies and reveals diverse evolutionary patterns. Cell Stress Chaperones 13: 127–142. 18759000 10.1007/s12192-008-0023-7 PMC2673885

[46] Dasgupta S, Hohman TC, Carper D (1992) Hypertonic stress induces alpha B-crystallin expression. Exp Eye Res 54: 461–470. 1381680 10.1016/0014-4835(92)90058-z

[47] Wang HS, Wang XH, Zhou CS, Huang LH, Zhang SF, et al (2007) cDNA cloning of heat shock proteins and their expression in the two phases of the migratory locust. Insect Mol Biol 16: 207–219. 17298555 10.1111/j.1365-2583.2006.00715.x

[48] Sonoda S, Fukumoto K, Izumi Y, Yoshida H, Tsumuki H (2006) Cloning of heat shock protein genes (hsp90 and hsc70) and their expression during larval diapause and cold tolerance acquisition in the rice stem borer, *Chilo suppressalis Walker* . Arch Insect Biochem Physiol 63: 36–47. 16921518 10.1002/arch.20138

[49] Zhang Q, Denlinger DL (2010) Molecular characterization of heat shock protein 90, 70 and 70 cognate cDNAs and their expression patterns during thermal stress and pupal diapause in the corn earworm. J Insect Physiol 56: 138–150. 19782689 10.1016/j.jinsphys.2009.09.013

[50] Rinehart JP, Li A, Yocum GD, Robich RM, Hayward SA, et al (2007) Up-regulation of heat shock proteins is essential for cold survival during insect diapause. Proc Natl Acad Sci U S A 104: 11130–11137. 17522254 10.1073/pnas.0703538104 PMC2040864

[51] van Heusden GP (2005) 14-3-3 proteins: regulators of numerous eukaryotic proteins. Iubmb Life 57: 623–629. 16203681 10.1080/15216540500252666

[52] Darling DL, Yingling J, Wynshaw-Boris A (2005) Role of 14-3-3 proteins in eukaryotic signaling and development. Curr Top Dev Biol 68: 281–315. 16125003 10.1016/S0070-2153(05)68010-6

[53] Feng Y, Qi W, Martinez J, Nelson MA (2005) The cyclin-dependent kinase 11 interacts with 14-3-3 proteins. Biochem Biophys Res Commun 331: 1503–1509. 15883043 10.1016/j.bbrc.2005.04.078

[54] Gao Y, Jiang M, Yang T, Ni J, Chen J (2006) A Cdc2-related protein kinase hPFTAIRE1 from human brain interacting with 14-3-3 proteins. Cell Research 16: 539–547. 16775625 10.1038/sj.cr.7310071

[55] Gurusamy N, Watanabe K, Ma M, Prakash P, Hirabayashi K, et al (2006) Glycogen synthase kinase 3beta together with 14-3-3 protein regulates diabetic cardiomyopathy: effect of losartan and tempol. FEBS Lett 580: 1932–1940. 16530186 10.1016/j.febslet.2006.02.056

[56] Dunaway S, Liu HY, Walworth NC (2005) Interaction of 14-3-3 protein with Chk1 affects localization and checkpoint function. Journal of Cell Science 118: 39–50. 15585577 10.1242/jcs.01570

[57] Hsu YC, Chern JJ, Cai Y, Liu M, Choi KW (2007) Drosophila TCTP is essential for growth and proliferation through regulation of dRheb GTPase. Nature 445: 785–788. 17301792 10.1038/nature05528

[58] Coppolino MG, Dedhar S (1998) Calreticulin. Int J Biochem Cell Biol 30: 553–558. 9693955 10.1016/s1357-2725(97)00153-2

[59] Goo TW, Park S, Jin BR, Yun EY, Kim I, et al (2005) Endoplasmic reticulum stress response of *Bombyx mori* calreticulin. Molecular Biology Reports 32: 133–139. 16172913 10.1007/s11033-004-5908-7

[60] Yamamoto Y, Watabe S, Kageyama T, Takahashi SY (1999) Purification and characterization of *Bombyx* cysteine proteinase specific inhibitors from the hemolymph of *Bombyx mori* . Arch Insect Biochem Physiol 42: 119–129. 10504205 10.1002/(SICI)1520-6327(199910)42:2<119::AID-ARCH2>3.0.CO;2-C

[61] Yoshiga T, Yokoyama N, Imai N, Ohnishi A, Moto K, et al (2002) cDNA cloning of calcineurin heterosubunits from the pheromone gland of the silkmoth, *Bombyx mori* . Insect Biochemistry and Molecular Biology 32: 477–486. 11886782 10.1016/s0965-1748(01)00125-4

[62] Somarelli JA, Herrera RJ (2007) Evolution of the 12 kDa FK506-binding protein gene. Biology of the Cell 99: 311–321. 17309448 10.1042/BC20060125

[63] Siva AB, Kameshwari DB, Singh V, Pavani K, Sundaram CS, et al (2010) Proteomics-based study on asthenozoospermia: differential expression of proteasome alpha complex. Mol Hum Reprod 16: 452–462. 20304782 10.1093/molehr/gaq009

[64] Quan G-X, Mita K, Okano K, Shimada T, Nanako U, et al (2001) Isolation and expression of the ecdysteroid-inducible angiotensin-converting enzyme-related gene in wing discs of *Bombyx mori* . Insect Biochemistry and Molecular Biology 31: 97–103. 11102839 10.1016/s0965-1748(00)00112-0

[65] Consortium TGO (2010) The Gene Ontology in 2010: extensions and refinements. Nucleic Acids Res 38: D331–335. 19920128 10.1093/nar/gkp1018 PMC2808930

[66] Hunter S, Apweiler R, Attwood TK, Bairoch A, Bateman A, et al (2009) InterPro: the integrative protein signature database. Nucleic Acids Res 37: D211–215. 18940856 10.1093/nar/gkn785 PMC2686546

[67] Sahoo SS, Bodenreider O, Rutter JL, Skinner KJ, Sheth AP (2008) An ontology-driven semantic mashup of gene and biological pathway information: Application to the domain of nicotine dependence. Journal of Biomedical Informatics 41: 752–765. 18395495 10.1016/j.jbi.2008.02.006 PMC2766186

[68] Yarden Y, Sliwkowski MX (2001) Untangling the ErbB signalling network. Nature Reviews Molecular Cell Biology 2: 127–137. 11252954 10.1038/35052073

[69] Shilo BZ (2003) Signaling by the Drosophila epidermal growth factor receptor pathway during development. Experimental Cell Research 284: 140–149. 12648473 10.1016/s0014-4827(02)00094-0

