# Supplementary material for: Shotgun Proteomic Analysis on the Diapause and Non-Diapause Eggs of Domesticated Silkworm Bombyx mori
Source: PLoS One. 2013 Apr 8;8(4):e60386. doi: 10.1371/journal.pone.0060386 (PMC3620277; doi:10.1371/journal.pone.0060386)
Supplement: Table S1 — The common-expressed proteins of D and ND with functional annotation. (DOC) [file pone.0060386.s001.doc]

Table S1 the common-expressed proteins of D and ND with functional annotation

| **Gene ID/**GI number | Theor.pI/ Mw(kDa) | No. of peptides^☆^ | No. of unique peptides^☆^ | Cover percent^☆^(%) | Protein description |
| --- | --- | --- | --- | --- | --- |
| gi|187281695| | 6.14/63299.82 | 523 / 621 | 32 / 29 | 75.85 / 72.27 | egg-specific protein |
| gi|156119320| | 6.83/30034.89 | 339 / 281 | 24 / 24 | 69.70 / 70.08 | low molecular lipoprotein 30K precursor |
| gi|162461355| | 6.83/30013.96 | 309 / 263 | 19 / 20 | 62.07 / 62.45 | low molecular lipoprotein 30K precursor |
| BGIBMGA004394 gi|112984502| | 6.11/29733.86 | 107 / 146 | 10 / 13 | 46.09 / 58.20 | low molecular lipoprotein 30K precursor |
| gi|112983746| | 6.85/203053.95 | 1274 /1120 | 96 / 91 | 65.88 / 64.31 | vitellogenin precursor |
| BGIBMGA004612 gi|112983556| | 4.99/82422.39 | 40 / 46 | 20 / 23 | 30.17 / 33.94 | 90-kDa heat shock protein |
| gi|148298693| | 5.98/20804.5 | 41 / 43 | 13 / 14 | 57.53 / 58.06 | heat shock protein hsp20.8 |
| BGIBMGA004541 gi|112983152| | 6.54/20427.15 | 41 / 42 | 13 / 14 | 67.96 / 73.48 | heat shock protein hsp20.4 |
| gi|112983134| | 5.46/20138.61 | 18 / 15 | 8 / 9 | 44.94 / 48.88 | heat shock protein hsp20.1 |
| BGIBMGA004540 gi|112983420| | 6.53/19890.56 | 29 / 27 | 7 / 8 | 55.93 / 59.89 | heat shock protein hsp 19.9 |
| gi|112983144| | 5.21/23570.74 | 8 / 5 | 4 / 3 | 16.27 / 12.92 | heat shock protein hsp23.7 |
| BGIBMGA000944 gi|112983414| | 5.79/21404.01 | 5 / 6 | 3 / 3 | 25.13 / 29.95 | heat shock protein hsp21.4 |
| gi|113676411| | 5.47/73585.32 | 31 / 19 | 12 / 11 | 22.04 / 20.86 | heat shock protein 70 |
| BGIBMGA002381 gi|112982828| | 5.33/71175.3 | 30 / 35 | 12 / 19 | 25.42 / 38.98 | heat shock cognate protein |
| BGIBMGA004375 gi|112982844| | 10.68/31433.85 | 1 / 6 | 1 / 5 | 4.09 / 24.16 | ribosomal protein L7 |
| BGIBMGA007661 gi|112983462| | 10.52/30292.89 | 1 / 2 | 1 / 2 | 4.48 / 7.84 | ribosomal protein L7A |
| BGIBMGA010571 gi|112983495| | 9.94/21377.03 | 8 / 7 | 5 / 5 | 34.74 / 29.47 | ribosomal protein L9 |
| BGIBMGA003337 gi|112983523| | 9.76/24589.92 | 1 / 1 | 1 / 1 | 5.99 / 5.99 | ribosomal protein L10A |
| BGIBMGA008881 gi|112984334| | 10.18/22326.94 | 5 / 3 | 3 / 2 | 15.90 / 11.79 | ribosomal protein L11 |
| BGIBMGA003726 gi|112983562| | 11.37/25139.48 | 2 / 4 | 1 / 2 | 5.91 / 10.45 | ribosomal protein L13 |
| BGIBMGA008865 gi|112984370| | 10.95/23357.59 | 2 / 2 | 1 / 2 | 7.35 / 10.78 | ribosomal protein L13A |
| BGIBMGA006414 gi|112984404| | 11.83/24038.03 | 3 / 6 | 2 / 3 | 12.25 / 16.67 | ribosomal protein L15 |
| BGIBMGA012414 gi|112984422| | 10.36/21598.12 | 2 / 6 | 2 / 3 | 5.88 / 11.23 | ribosomal protein L17 |
| BGIBMGA011620 gi|112984318| | 11.75/20864.56 | 3 / 2 | 2 / 1 | 14.21 / 3.83 | ribosomal protein L18 |
| BGIBMGA002572 gi|112984306| | 11.23/23610.87 | 2 / 2 | 1 / 1 | 5.00 / 5.00 | ribosomal protein L19 |
| BGIBMGA006835 gi|112984266| | 10.91/35964.53 | 5 / 4 | 3 / 2 | 13.92 / 9.66 | ribosomal protein L23A |
| BGIBMGA005684 gi|112982751| | 10.15/8240.89 | 2 / 2 | 1 / 1 | 17.14 / 17.14 | ribosomal protein L38 |
| BGIBMGA009319 gi|112984112| | 9.64/26771.22 | 1 / 3 | 1 / 3 | 5.35 / 14.81 | ribosomal protein S3 |
| BGIBMGA007710 gi|112984070| | 9.39/24538.22 | 3 / 5 | 2 / 3 | 12.79 / 16.89 | ribosomal protein S5 |
| BGIBMGA004356 gi|112984058| | 9.82/21858.54 | 1 / 3 | 1 / 3 | 14.21 / 14.21 | ribosomal protein S7 |
| BGIBMGA003397 gi|112984034| | 10.76/23791.45 | 10 / 7 | 6 / 4 | 36.06 / 23.56 | ribosomal protein S8 |
| BGIBMGA012626 gi|112984022| | 10.76/22576.42 | 2 / 8 | 2 / 5 | 6.19 / 16.49 | ribosomal protein S9 |
| BGIBMGA006867 gi|112982855| | 10.01/14692.29 | 2 / 2 | 2 / 1 | 17.05 / 10.85 | ribosomal protein S15A |
| BGIBMGA010139 gi|112983932| | 9.91/9433.12 | 2 / 2 | 1 / 2 | 11.90 / 21.43 | ribosomal protein S27 |
| BGIBMGA011948 gi|112982910| | 10.38/7318.47 | 4 / 2 | 2 / 1 | 32.31 / 18.46 | ribosomal protein S28 |
| BGIBMGA011446 gi|112983904| | 10.12/14295.33 | 2 / 2 | 1 / 1 | 8.46 / 7.69 | ribosomal protein S30 |
| BGIBMGA010794 gi|114052605| | 7.03/44777.77 | 2 / 1 | 1 / 1 | 2.78 / 3.54 | 26S proteasome regulatory ATPase subunit 10B |
| BGIBMGA002005 gi|158631166| | 9.88/32891.13 | 2 / 5 | 1 / 3 | 3.33 / 12.67 | ADP/ATP translocase |
| BGIBMGA005812 gi|112983926| | 5.87/39991.31 | 21 / 15 | 8 / 9 | 31.83 / 34.37 | arginine kinase |
| BGIBMGA001853 gi|114052278| | 9.21/59657.9 | 12 / 14 | 7 / 8 | 17.18 / 20.07 | ATP synthase |
| BGIBMGA012630 gi|113865941| | 7.22/44236.75 | 2 / 1 | 1 / 1 | 4.40 / 2.59 | ecdysteroid 22-kinase |
| BGIBMGA011342 gi|164420679| | 5.9/38138.83 | 7 / 10 | 4 / 6 | 14.96 / 22.29 | fibroinase |
| BGIBMGA007438 gi|114051481| | 5.93/47373.06 | 7 / 8 | 4 / 5 | 11.35 / 14.18 | fumarylacetoacetate hydrolase |
| BGIBMGA013021 gi|148298685| | 8.38/39653.3 | 5 / 12 | 3 / 6 | 10.99 / 25.55 | fructose 1,6-bisphosphate aldolase |
| BGIBMGA004221 gi|148298746| | 6.45/61982.5 | 4 / 6 | 2 / 5 | 4.68 / 13.13 | glucose-6-phosphate isomerase |
| BGIBMGA006507 gi|114052462| | 8.36/61397.42 | 3 / 6 | 2 / 4 | 4.69 / 11.01 | glutamate dehydrogenase |
| BGIBMGA002222 gi|112983444| | 7.72/24226.82 | 10 / 9 | 5 / 6 | 29.63 / 36.11 | glutathione S-transferase delta |
| BGIBMGA009107 gi|160333678| | 5.85/23339.74 | 9 / 10 | 5 / 5 | 28.43 / 30.39 | glutathione S-transferase sigma |
| BGIBMGA012549 gi|114052072| | 5.32/54835.83 | 14 / 15 | 9 / 8 | 23.06 / 19.38 | H+ transporting ATP synthase beta subunit isoform 1 |
| BGIBMGA011936 gi|153791739| | 5.56/20202.91 | 1 / 2 | 1 / 1 | 7.82 / 7.82 | H+ transporting ATP synthase subunit d |
| BGIBMGA013511 gi|114051097| | 9.93/10924.9 | 3 / 2 | 2 / 1 | 24.24 / 8.08 | H+ transporting ATP synthase subunit g |
| gi|114053277| | 9.66/22501.36 | 3 / 3 | 2 / 2 | 9.09 / 10.53 | H+ transporting ATP synthase subunit O |
| BGIBMGA002967 gi|114052230| | 9.38/82141.33 | 1 / 8 | 1 / 6 | 2.10 / 14.83 | hydroxyacyl-coenzyme A dehydrogenase |
| BGIBMGA006907 gi|114051866| | 6.24/46176.05 | 5 / 6 | 1 / 3 | 3.19 / 10.05 | isocitrate dehydrogenase |
| BGIBMGA009614 gi|114051964| | 8.41/25599.71 | 5 / 6 | 4 / 3 | 22.37 / 17.54 | lysosomal thiol reductase IP30 isoform 1 |
| BGIBMGA010403 gi|114051966| | 5.57/52761.28 | 24 / 20 | 14 / 11 | 41.19 / 35.45 | mitochondrial aldehyde dehydrogenase |
| gi|114052408| | 7.52/55849.47 | 5 / 3 | 3 / 2 | 7.41 / 5.46 | mitochondrial aldehyde dehydrogenase |
| BGIBMGA007453 gi|112983802| | 8.84/24226.46 | 6 / 6 | 4 / 4 | 24.07 / 28.70 | Mn superoxide dismutase |
| BGIBMGA004059 gi|114052472| | 7.88/22396.5 | 10 / 15 | 7 / 7 | 39.51 / 36.59 | peptidylprolyl isomerase B |
| gi|163838668| | 5.67/80181.61 | 13 / 12 | 9 / 8 | 15.30 / 13.13 | prophenoloxidase-2s |
| BGIBMGA012763 gi|112983667| | 6.25/78785.37 | 2 / 3 | 1 / 2 | 1.90 / 4.38 | prophenoloxidase subunit 1 |
| BGIBMGA011844 gi|112984454| | 4.6/55588.87 | 5 / 11 | 3 / 7 | 9.72 / 19.03 | protein disulfide isomerase |
| BGIBMGA003587 gi|112983366| | 5.3/55087.31 | 7 / 11 | 5 / 9 | 12.42 / 24.64 | protein disulfide-isomerase like protein ERp57 |
| BGIBMGA011168 gi|153791817| | 5.79/47483.67 | 7 / 3 | 4 / 2 | 12.09 / 6.28 | S-adenosyl-L-homocysteine hydrolase |
| BGIBMGA004091 gi|114050819| | 5.5/11220.24 | 1 / 2 | 1 / 1 | 13.00 / 13.00 | salivary secreted ribonuclease |
| BGIBMGA002186 gi|112982996| | 6.09/21916.03 | 26 / 21 | 10 / 9 | 48.72 / 41.54 | thiol peroxiredoxin |
| BGIBMGA000064 gi|114052210| | 8.33/25283.89 | 2 / 2 | 1 / 1 | 5.73 / 5.73 | thioredoxin peroxidase |
| gi|114050833| | 6.4/67384.01 | 21 / 12 | 13 / 9 | 29.90 / 20.26 | transketolase |
| BGIBMGA003985 gi|112983322| | 5.3/89152.91 | 4 / 10 | 4 / 8 | 6.34 / 16.02 | transitional endoplasmic reticulum ATPase TER94 |
| BGIBMGA000559 gi|187281708| | 5.67/26778.49 | 8 / 5 | 4 / 3 | 22.18 / 15.32 | triosephosphate isomerase |
| gi|148298878| | 5.27/68315.93 | 6 / 11 | 4 / 8 | 10.21 / 17.83 | vacuolar ATP synthase catalytic subunit A |
| BGIBMGA000596 gi|114052663| | 6.15/55130.49 | 3 / 1 | 2 / 1 | 6.11 / 2.74 | vacuolar ATP synthase subunit H |
| BGIBMGA002241 gi|148298717| | 5.25/54416.37 | 2 / 7 | 2 / 6 | 7.55 / 20.41 | vacuolar ATP synthase subunit B |
| BGIBMGA013201 gi|148298752| | 4.66/29671.21 | 9 / 11 | 4 / 8 | 20.99 / 32.44 | 14-3-3 epsilon protein |
| gi|187281844| | 5.3/41821.89 | 27 / 18 | 11 / 10 | 37.77 / 34.57 | Actin A4 |
| BGIBMGA002906 gi|112983208| | 7.95/9912.36 | 1 / 1 | 1 / 1 | 9.89 / 9.89 | acyl-CoA binding protein |
| BGIBMGA002626 gi|164448664| | 6.71/15489.75 | 1 / 4 | 1 / 2 | 7.86 / 9.29 | antennal binding protein |
| BGIBMGA009953 gi|112983770| | 5.41/43498.53 | 1 / 13 | 1 / 10 | 2.81 / 36.26 | antitrypsin |
| BGIBMGA010644 gi|166706856| | 9.45/101338.23 | 2 / 2 | 1 / 2 | 1.56 / 3.34 | Aubergine protein |
| BGIBMGA009132 gi|112983318| | 4.75/50214.47 | 17 / 26 | 7 / 14 | 27.29 / 49.66 | beta-tubulin |
| gi|112983456| | 4.77/51353.7 | 9 / 11 | 5 / 6 | 15.10 / 21.44 | beta-tubulin |
| BGIBMGA012710 gi|114051135| | 5.06/26330.01 | 1 / 1 | 1 / 1 | 9.83 / 9.83 | BWK-1-like protein |
| BGIBMGA000475 gi|112983032| | 4.49/45801.94 | 2 / 3 | 1 / 2 | 4.52 / 7.79 | calreticulin |
| BGIBMGA009790 gi|112983368| | 7.58/54154.8 | 1 / 1 | 1 / 1 | 2.73 / 2.73 | carotenoid-binding protein isoform 1 |
| gi|112983600| | 5.66/14858.88 | 6 / 3 | 4 / 2 | 36.36 / 16.67 | cellular retinoic acid binding protein |
| gi|120444903| | 5.4/59173.91 | 2 / 3 | 1 / 2 | 2.94 / 4.77 | chaperonin |
| BGIBMGA001206 gi|114051313| | 6.32/57590.41 | 11 / 18 | 7 / 11 | 19.96 / 30.22 | chaperonin containing t-complex polypeptide 1 beta subunit |
| BGIBMGA010666 gi|114052545| | 6.9/56645.4 | 8 / 8 | 5 / 5 | 12.85 / 18.25 | chaperonin subunit 4 delta |
| BGIBMGA013116 gi|114050749| | 6.59/57744.83 | 6 / 5 | 4 / 4 | 9.79 / 10.55 | chaperonin subunit 6a zeta |
| BGIBMGA004041 gi|112983052| | 4.97/13519.5 | 1 / 2 | 1 / 2 | 9.84 / 18.85 | chemosensory protein 11 |
| gi|187281701| | 8.16/9544.02 | 3 / 2 | 2 / 1 | 12.94 / 12.94 | chymotrypsin inhibitor CI-b1 |
| BGIBMGA008205 gi|112983016| | 8.82/9633.5 | 3 / 1 | 2 / 1 | 25.84 / 12.36 | CP8 precursor |
| BGIBMGA005274 gi|114052957| | 8.57/19905.98 | 1 / 1 | 1 / 1 | 5.52 / 6.08 | dynactin 4 protein |
| BGIBMGA007230 gi|114053033| | 5.16/66038.49 | 3 / 2 | 2 / 1 | 3.98 / 2.65 | Ef1alpha-like factor isoform 1 |
| BGIBMGA008921 gi|112982743| | 4.49/24548.52 | 3 / 5 | 2 / 4 | 13.06 / 22.97 | elongation factor 1 beta |
| BGIBMGA008302 gi|112983898| | 5.84/48388 | 9 / 6 | 4 / 5 | 9.22 / 14.89 | elongation factor 1 gamma |
| BGIBMGA003669 gi|112983938| | 5.54/52114.33 | 2 / 2 | 2 / 1 | 6.52 / 4.27 | eukaryotic translation initiation factor 3 subunit 6 |
| BGIBMGA013996 gi|114053141| | 7.11/45807.02 | 1 / 2 | 1 / 1 | 5.81 / 5.81 | exuperantia |
| BGIBMGA008768 gi|112982932| | 5.43/23390.82 | 8 / 14 | 4 / 8 | 15.31 / 45.93 | ferritin |
| BGIBMGA006751 gi|114052751| | 6.96/24336.89 | 5 / 6 | 3 / 4 | 17.84 / 23.00 | GTP-binding nuclear protein Ran |
| BGIBMGA005115 gi|112983246| | 5.44/22364.31 | 6 / 7 | 4 / 5 | 22.28 / 27.72 | GTP-binding protein |
| BGIBMGA006945 gi|114053313| | 7.09/44474.1 | 2 / 4 | 1 / 2 | 0.99 / 1.86 | GTP binding protein |
| BGIBMGA001928 gi|112983258| | 6.23/23294.29 | 2 / 4 | 1 / 3 | 6.54 / 17.76 | GTP-binding protein RAB2 |
| BGIBMGA013449 gi|114053181| | 10.55/13372.49 | 4 / 3 | 2 / 2 | 12.40 / 12.40 | H2A histone family member V |
| BGIBMGA001324 gi|112983174| | 7/26666.11 | 3 / 1 | 2 / 1 | 8.90 / 5.51 | hypothetical protein LOC692495 |
| BGIBMGA008174 gi|114052390| | 7.04/27982.71 | 4 / 6 | 3 / 3 | 14.90 / 17.25 | insulin-related peptide binding protein |
| BGIBMGA003758 gi|114053323| | 4.85/56500.85 | 1 / 1 | 1 / 1 | 1.74 / 2.91 | karyopherin alpha 3 |
| BGIBMGA011573 gi|114052484| | 8.82/10582.3 | 2 / 2 | 1 / 1 | 13.54 / 13.54 | kazal-type proteinase inhibitor |
| BGIBMGA006809  gi|182511222| | 5.82/108295.6 | 1 / 3 | 1 / 2 | 1.24 / 3.01 | kinesin heavy chain |
| BGIBMGA000086 gi|114051600| | 7.52/30640.18 | 4 / 8 | 3 / 5 | 18.18 / 21.45 | LRP16 protein |
| BGIBMGA009841 gi|114052587| | 5.13/31974.92 | 4 / 1 | 2 / 1 | 12.54 / 4.88 | mitochondrial matrix protein p33 |
| BGIBMGA007266 gi|114050829| | 6.82/13439.45 | 1 / 2 | 1 / 1 | 10.40 / 10.40 | mitogen-activated protein-binding protein-interacting protein |
| BGIBMGA000381 gi|114052643| | 7.64/38001.61 | 1 / 2 | 1 / 1 | 3.68 / 3.68 | nuclear migration protein nudC |
| BGIBMGA009249 gi|114052018| | 4.51/43045.91 | 4 / 7 | 2 / 4 | 6.38 / 11.97 | nucleosome assembly protein |
| BGIBMGA000462 gi|112983548| | 5.12/24902.49 | 10 / 11 | 7 / 5 | 30.40 / 28.19 | p27K |
| BGIBMGA013593 gi|114051003| | 8.89/41546.87 | 17 / 12 | 9 / 7 | 33.24 / 29.76 | perilipin |
| BGIBMGA010604 gi|112983712| | 9.74/14796.3 | 6 / 3 | 3 / 1 | 24.03 / 10.08 | ubiquitin/ribosomal protein L40 fusion protein |
| BGIBMGA002981 gi|112982865| | 5.88/13712.67 | 8 / 6 | 5 / 3 | 65.08 / 39.68 | profilin |
| BGIBMGA006158 gi|114053221| | 6.45/30081.56 | 3 / 5 | 2 / 4 | 13.50 / 28.10 | prohibitin protein WPH |
| BGIBMGA009562 gi|114052504| | 5.88/25824.45 | 1 / 4 | 1 / 3 | 8.12 / 18.38 | proteasome 25 kDa subunit |
| BGIBMGA007268 gi|114052086| | 6.14/51814.55 | 1 / 2 | 1 / 1 | 2.89 / 2.89 | proteasome 26S non-ATPase subunit 12 |
| gi|114051245| | 5.27/28258.04 | 2 / 1 | 1 / 1 | 4.31 / 4.31 | proteasome alpha 3 subunit |
| BGIBMGA000201 gi|151301141| | 5.97/25756.35 | 6 / 4 | 3 / 2 | 13.36 / 9.05 | proteasome beta subunit |
| BGIBMGA003597 gi|114052691| | 5.53/23628.66 | 4 / 4 | 2 / 2 | 10.53 / 10.53 | RAB6A, member RAS oncogene family |
| BGIBMGA007712 gi|114051368| | 5.16/23420.43 | 9 / 5 | 6 / 4 | 32.69 / 23.08 | Rab7 |
| BGIBMGA002209 gi|112983314| | 5.24/24092.12 | 9 / 9 | 5 / 6 | 24.88 / 31.46 | ras-related GTP-binding protein Rab11 |
| BGIBMGA007311 gi|162952033| | 4.87/33408.77 | 2 / 3 | 2 / 2 | 10.46 / 10.46 | ribosome-associated protein P40 |
| BGIBMGA012451 gi|112983434| | 8.36/24355.41 | 2 / 6 | 1 / 3 | 6.02 / 16.20 | SCP-related protein |
| BGIBMGA006045 gi|112983262| | 8.73/23173.26 | 4 / 7 | 2 / 5 | 11.74 / 29.11 | small GTP binding protein RAB5 |
| BGIBMGA004666 gi|148298847| | 8.3/23125.54 | 5 / 4 | 3 / 3 | 17.24 / 16.75 | small GTP-binding protein Rab10 |
| BGIBMGA009103 gi|112983786| | 6.54/57879.03 | 1 / 1 | 1 / 1 | 2.80 / 2.05 | sterol carrier protein x |
| BGIBMGA011334 gi|114052645| | 4.95/19016.23 | 8 / 10 | 5 / 6 | 34.12 / 41.76 | thymosin isoform 1 |
| BGIBMGA011424 gi|112983240| | 6.89/75722.56 | 2 / 4 | 2 / 4 | 3.08 / 6.75 | transferrin |
| gi|112982880| | 4.66/19859.74 | 3 / 8 | 3 / 4 | 13.37 / 25.58 | translationally controlled tumor protein |
| gi|153792203| | 5.85/48820.41 | 7 / 9 | 5 / 6 | 14.29 / 22.32 | vesicle amine transport protein |

^☆^stands for the parameters of D and ND
