# Supplementary material for: Shotgun Proteomic Analysis on the Diapause and Non-Diapause Eggs of Domesticated Silkworm Bombyx mori
Source: PLoS One. 2013 Apr 8;8(4):e60386. doi: 10.1371/journal.pone.0060386 (PMC3620277; doi:10.1371/journal.pone.0060386)
Supplement: Table S2 — KEGG pathways of all identified proteins. (DOC) [file pone.0060386.s002.doc]

Table S2 KEGG pathways of all identified proteins

| **Annotation** |  | **Total**  **Genes** | **D-unique Genes** | | **N-unique**  **Genes** | **D and N Common Genes** |
| --- | --- | --- | --- | --- | --- | --- |
| **Metabolism** |  |  |  | |  |  |
| Carbohydrate Metabolism | Glycolysis / Gluconeogenesis | 211 | 40 | | 60 | 111 |
|  | Citrate cycle (TCA cycle) | 78 | 0 | | 20 | 58 |
|  | Pentose phosphate pathway | 132 | 21 | | 32 | 79 |
|  | Pentose and glucuronate interconversions | 116 | 13 | | 30 | 73 |
|  | Fructose and mannose metabolism | 82 | 33 | | 29 | 20 |
|  | Galactose metabolism | 69 | 29 | | 20 | 20 |
|  | Ascorbate and aldarate metabolism | 43 | 1 | | 8 | 34 |
|  | Starch and sucrose metabolism | 113 | 20 | | 33 | 60 |
|  | Amino sugar and nucleotide sugar metabolism | 94 | 20 | | 34 | 40 |
|  | Inositol phosphate metabolism | 40 | 20 | | 20 | 0 |
|  | Glyoxylate and dicarboxylate metabolism | 20 | 0 | | 20 | 0 |
|  | Propanoate metabolism | 79 | 19 | | 8 | 52 |
|  | Pyruvate metabolism | 114 | 9 | | 34 | 71 |
|  | Butanoate metabolism | 37 | 19 | | 17 | 1 |
| Lipid Metabolism | Fatty acid biosynthesis | 18 | 0 | | 18 | 0 |
|  | Fatty acid elongation in mitochondria | 57 | 19 | | 0 | 38 |
|  | Fatty acid metabolism | 153 | 38 | | 25 | 90 |
|  | Synthesis and degradation of ketone bodies | 1 | 0 | | 0 | 1 |
|  | Primary bile acid biosynthesis | 20 | 0 | | 0 | 20 |
|  | Steroid hormone biosynthesis | 3 | 3 | | 0 | 0 |
|  | Glycerolipid metabolism | 78 | 29 | | 15 | 34 |
|  | Glycerophospholipid metabolism | 40 | 20 | | 20 | 0 |
|  | Arachidonic acid metabolism | 51 | 0 | | 51 | 0 |
|  | Linoleic acid metabolism | 3 | 3 | | 0 | 0 |
|  | Biosynthesis of unsaturated fatty acids | 14 | 14 | | 0 | 0 |
| Nucleotide Metabolism | Purine metabolism | 234 | 88 | | 90 | 56 |
|  | Pyrimidine metabolism | 119 | 57 | | 62 | 0 |
| Amino Acid Metabolism | Alanine, aspartate and glutamate metabolism | 94 | 0 | | 38 | 56 |
|  | Glycine, serine and threonine metabolism | 42 | 20 | | 20 | 2 |
|  | Cysteine and methionine metabolism | 59 | 19 | | 0 | 40 |
|  | Valine, leucine and isoleucine degradation | 134 | 19 | | 25 | 90 |
|  | Valine, leucine and isoleucine biosynthesis | 39 | 20 | | 0 | 19 |
|  | Lysine degradation | 108 | 19 | | 35 | 54 |
|  | Arginine and proline metabolism | 148 | 0 | | 57 | 91 |
|  | Histidine metabolism | 48 | 0 | | 14 | 34 |
|  | Tyrosine metabolism | 25 | 0 | | 6 | 19 |
|  | Phenylalanine metabolism | 6 | 0 | | 6 | 0 |
|  | Tryptophan metabolism | 86 | 19 | | 14 | 53 |
| Metabolism of Other Amino Acids | beta-Alanine metabolism | 136 | 56 | | 28 | 52 |
|  | Taurine and hypotaurine metabolism | 34 | 19 | | 15 | 0 |
|  | Selenocompound metabolism | 40 | 0 | | 40 | 0 |
|  | Cyanoamino acid metabolism | 35 | 0 | | 35 | 0 |
|  | D-Glutamine and D-glutamate metabolism | 20 | 0 | | 0 | 20 |
|  | Glutathione metabolism | 157 | 16 | | 70 | 71 |
| Glycan Biosynthesis and Metabolism | N-Glycan biosynthesis | 39 | 0 | | 19 | 20 |
|  | Glycosaminoglycan degradation | 16 | 1 | | 0 | 15 |
|  | Various types of N-glycan biosynthesis | 20 | 0 | | 0 | 20 |
| Xenobiotics Biodegradation and Metabolism | Chloroalkane and chloroalkene degradation | 42 | 0 | | 8 | 34 |
|  | Aminobenzoate degradation | 37 | 22 | | 15 | 0 |
|  | Styrene degradation | 19 | 0 | | 0 | 19 |
|  | Benzoate degradation | 57 | 19 | | 0 | 38 |
|  | Caprolactam degradation | 20 | 20 | | 0 | 0 |
|  | Drug metabolism - cytochrome P450 | 47 | 14 | | 0 | 33 |
|  | Metabolism of xenobiotics by cytochrome P450 | 47 | 14 | | 0 | 33 |
|  | Drug metabolism - other enzymes | 80 | 40 | | 20 | 20 |
| Energy Metabolism | Methane metabolism | 76 | 0 | | 58 | 18 |
|  | Carbon fixation in photosynthetic organisms | 137 | 20 | | 39 | 78 |
|  | Carbon fixation pathways in prokaryotes | 38 | 0 | | 0 | 38 |
|  | Oxidative phosphorylation | 303 | 40 | | 79 | 184 |
|  | Nitrogen metabolism | 59 | 20 | | 0 | 39 |
| Metabolism of Cofactors and Vitamins | Riboflavin metabolism | 1 | 0 | | 0 | 1 |
|  | One carbon pool by folate | 45 | 20 | | 25 | 0 |
|  | Nicotinate and nicotinamide metabolism | 22 | 20 | | 2 | 0 |
|  | Pantothenate and CoA biosynthesis | 57 | 37 | | 20 | 0 |
|  | Folate biosynthesis | 74 | 40 | | 15 | 19 |
|  | Retinol metabolism | 11 | 3 | | 8 | 0 |
|  | Porphyrin and chlorophyll metabolism | 39 | 0 | | 20 | 19 |
|  | Ubiquinone and other terpenoid-quinone biosynthesis | 19 | 0 | | 19 | 0 |
| Metabolism of Terpenoids and Polyketides | Terpenoid backbone biosynthesis | 20 | 0 | | 20 | 0 |
|  | Limonene and pinene degradation | 42 | 0 | | 8 | 34 |
|  | Isoquinoline alkaloid biosynthesis | 6 | 0 | | 6 | 0 |
|  | Indole alkaloid biosynthesis | 6 | 0 | | 6 | 0 |
|  | Streptomycin biosynthesis | 33 | 20 | | 13 | 0 |
|  | Betalain biosynthesis | 6 | 0 | | 6 | 0 |
|  | Insect hormone biosynthesis | 37 | 0 | | 37 | 0 |
|  | Biosynthesis of ansamycins | 20 | 0 | | 0 | 20 |
| **Genetic Information Processing** |  |  |  | |  |  |
| Translation | Aminoacyl-tRNA biosynthesis | 185 | 80 | | 40 | 65 |
|  | RNA transport | 20 | 0 | | 20 | 0 |
|  | mRNA surveillance pathway | 20 | 0 | | 20 | 0 |
| Transcription | Spliceosome | 20 | 0 | | 20 | 0 |
| Replication and Repair | Homologous recombination | 19 | 0 | | 0 | 19 |
| Folding, Sorting and Degradation | Ubiquitin mediated proteolysis | 40 | 0 | | 20 | 20 |
|  | Sulfur relay system | 20 | 20 | | 0 | 0 |
|  | Protein processing in endoplasmic reticulum | 152 | 19 | | 35 | 98 |
|  | Proteasome | 177 | 20 | | 39 | 118 |
|  | RNA degradation | 58 | 20 | | 0 | 38 |
| **Environmental Information Processing** |  |  |  | |  |  |
| Signal Transduction | MAPK signaling pathway | 84 | 45 | | 20 | 19 |
|  | ErbB signaling pathway | 20 | 20 | | 0 | 0 |
|  | MAPK signaling pathway - fly | 39 | 20 | | 19 | 0 |
|  | Calcium signaling pathway | 98 | 58 | | 40 | 0 |
|  | Phosphatidylinositol signaling system | 20 | 20 | | 0 | 0 |
|  | Two-component system | 34 | 0 | | 15 | 19 |
| Signaling Molecules and Interaction | Cytokine-cytokine receptor interaction | 2 | 0 | | 2 | 0 |
|  | Neuroactive ligand-receptor interaction | 7 | 1 | | 6 | 0 |
| **Cellular Processes** |  |  |  | |  |  |
| Transport and Catabolism | Lysosome | 41 | 3 | | 0 | 38 |
|  | Endocytosis | 19 | 0 | | 19 | 0 |
|  | Phagosome | 119 | 0 | | 41 | 78 |
|  | Peroxisome | 108 | 19 | | 28 | 61 |
|  | Regulation of autophagy | 20 | 20 | | 0 | 0 |
| Cell Communication | Focal adhesion | 21 | 21 | | 0 | 0 |
|  | Tight junction | 14 | 0 | | 0 | 14 |
|  | Gap junction | 78 | 39 | | 39 | 0 |
| Cell Growth and Death | Cell cycle - Caulobacter | 20 | 20 | | 0 | 0 |
|  | Meiosis - yeast | 20 | 0 | | 20 | 0 |
|  | Oocyte meiosis | 79 | 59 | | 20 | 0 |
|  | Apoptosis | 40 | 20 | | 20 | 0 |
| Cell Motility | Regulation of actin cytoskeleton | 21 | 21 | | 0 | 0 |
| **Environmental Information Processing** |  |  |  | |  |  |
| Signal Transduction | Wnt signaling pathway | 41 | 21 | | 20 | 0 |
|  | Hedgehog signaling pathway | 20 | 0 | | 20 | 0 |
|  | TGF-beta signaling pathway | 3 | 1 | | 2 | 0 |
|  | mTOR signaling pathway | 20 | 20 | | 0 | 0 |
|  | VEGF signaling pathway | 40 | 40 | | 0 | 0 |
| **Organismal Systems** |  |  |  | |  |  |
| Circulatory System | Cardiac muscle contraction | 66 | 20 | | 19 | 27 |
|  | Vascular smooth muscle contraction | 103 | 50 | | 39 | 14 |
| Development | Dorso-ventral axis formation | 20 | 20 | | 0 | 0 |
|  | Axon guidance | 21 | 21 | | 0 | 0 |
|  | Osteoclast differentiation | 40 | 40 | | 0 | 0 |
| Environmental Adaptation | Plant-pathogen interaction | 20 | 20 | | 0 | 0 |
| Immune System | Toll-like receptor signaling pathway | 20 | 20 | | 0 | 0 |
|  | RIG-I-like receptor signaling pathway | 20 | 0 | | 20 | 0 |
|  | Complement and coagulation cascades | 9 | 9 | | 0 | 0 |
|  | Antigen processing and presentation | 39 | 0 | | 0 | 39 |
|  | Chemokine signaling pathway | 60 | 40 | | 20 | 0 |
|  | Hematopoietic cell lineage | 23 | 5 | | 0 | 18 |
|  | Natural killer cell mediated cytotoxicity | 40 | 40 | | 0 | 0 |
|  | T cell receptor signaling pathway | 40 | 40 | | 0 | 0 |
|  | B cell receptor signaling pathway | 40 | 40 | | 0 | 0 |
|  | Fc epsilon RI signaling pathway | 20 | 20 | | 0 | 0 |
|  | Fc gamma R-mediated phagocytosis | 39 | 20 | | 19 | 0 |
|  | Leukocyte transendothelial migration | 1 | 1 | | 0 | 0 |
| Nervous System | Long-term potentiation | 60 | 40 | | 20 | 0 |
|  | Neurotrophin signaling pathway | 39 | 20 | | 0 | 19 |
|  | Glutamatergic synapse | 78 | 39 | | 20 | 19 |
|  | Cholinergic synapse | 59 | 39 | | 20 | 0 |
|  | Long-term depression | 41 | 20 | | 21 | 0 |
| Sensory System | Olfactory transduction | 20 | 0 | | 20 | 0 |
|  | Taste transduction | 20 | 0 | | 20 | 0 |
|  | Phototransduction - fly | 20 | 0 | | 20 | 0 |
| Endocrine System | Renin-angiotensin system | 61 | 5 | | 19 | 37 |
|  | PPAR signaling pathway | 71 | 33 | | 0 | 38 |
|  | Insulin signaling pathway | 98 | 40 | | 58 | 0 |
|  | GnRH signaling pathway | 59 | 39 | | 20 | 0 |
|  | Progesterone-mediated oocyte maturation | 59 | 39 | | 20 | 0 |
|  | Melanogenesis | 59 | 39 | | 20 | 0 |
|  | Adipocytokine signaling pathway | 39 | 39 | | 0 | 0 |
| Excretory System | Aldosterone-regulated sodium reabsorption | 19 | 0 | | 19 | 0 |
|  | Endocrine and other factor-regulated calcium reabsorption | 58 | 0 | | 58 | 0 |
|  | Vasopressin-regulated water reabsorption | 21 | 0 | | 20 | 1 |
|  | Proximal tubule bicarbonate reclamation | 39 | 0 | | 19 | 20 |
|  | Collecting duct acid secretion | 79 | 0 | | 39 | 40 |
| Digestive System | Salivary secretion | 79 | 19 | | 60 | 0 |
|  | Gastric acid secretion | 58 | 19 | | 39 | 0 |
|  | Pancreatic secretion | 63 | 20 | | 43 | 0 |
|  | Carbohydrate digestion and absorption | 19 | 0 | | 19 | 0 |
|  | Protein digestion and absorption | 26 | 1 | | 25 | 0 |
|  | Bile secretion | 58 | 19 | | 39 | 0 |
|  | Mineral absorption | 38 | 0 | | 19 | 19 |
| **Human Diseases** |  |  | |  |  |  |
| Metabolic Diseases | Type II diabetes mellitus | 19 | 0 | | 0 | 19 |
|  | Type I diabetes mellitus | 18 | 0 | | 0 | 18 |
| Neurodegenerative Diseases | Alzheimer's disease | 206 | 60 | | 60 | 86 |
|  | Parkinson's disease | 186 | 40 | | 40 | 106 |
|  | Amyotrophic lateral sclerosis (ALS) | 41 | 20 | | 2 | 19 |
|  | Huntington's disease | 185 | 40 | | 40 | 105 |
|  | Prion diseases | 40 | 20 | | 20 | 0 |
| Infectious Diseases | Bacterial invasion of epithelial cells | 19 | 0 | | 19 | 0 |
|  | Vibrio cholerae infection | 118 | 0 | | 59 | 59 |
|  | Epithelial cell signaling in Helicobacter pylori infection | 98 | 0 | | 39 | 59 |
|  | Pathogenic Escherichia coli infection | 1 | 1 | | 0 | 0 |
|  | Shigellosis | 1 | 1 | | 0 | 0 |
|  | Chagas disease (American trypanosomiasis) | 38 | 0 | | 19 | 19 |
|  | Toxoplasmosis | 1 | 0 | | 1 | 0 |
|  | Amoebiasis | 20 | 0 | | 20 | 0 |
|  | Tuberculosis | 39 | 20 | | 0 | 19 |
|  | Hepatitis C | 19 | 19 | | 0 | 0 |
|  | Measles | 19 | 19 | | 0 | 0 |
|  | Influenza A | 66 | 40 | | 26 | 0 |
| Cancers | Pathways in cancer | 20 | 20 | | 0 | 0 |
|  | Colorectal cancer | 20 | 20 | | 0 | 0 |
|  | Renal cell carcinoma | 20 | 20 | | 0 | 0 |
|  | Pancreatic cancer | 20 | 20 | | 0 | 0 |
|  | Endometrial cancer | 20 | 20 | | 0 | 0 |
|  | Glioma | 20 | 20 | | 0 | 0 |
|  | Prostate cancer | 20 | 20 | | 0 | 0 |
|  | Thyroid cancer | 20 | 20 | | 0 | 0 |
|  | Melanoma | 20 | 20 | | 0 | 0 |
|  | Bladder cancer | 20 | 20 | | 0 | 0 |
|  | Chronic myeloid leukemia | 20 | 20 | | 0 | 0 |
|  | Acute myeloid leukemia | 20 | 20 | | 0 | 0 |
|  | Non-small cell lung cancer | 20 | 20 | | 0 | 0 |
| Immune System Diseases | Rheumatoid arthritis | 117 | 0 | | 39 | 78 |
| Cardiovascular Diseases | Hypertrophic cardiomyopathy (HCM) | 61 | 23 | | 19 | 19 |
|  | Dilated cardiomyopathy(DCM) | 42 | 22 | | 20 | 0 |
